# Integrated single-cell RNA sequencing analysis reveals distinct cellular and transcriptional modules associated with survival in lung cancer

**DOI:** 10.1038/s41392-021-00824-9

**Published:** 2022-01-14

**Authors:** Li Zhang, Yiming Zhang, Chengdi Wang, Ying Yang, Yinyun Ni, Zhoufeng Wang, Tingting Song, Menglin Yao, Zhiqiang Liu, Ningning Chao, Yongfeng Yang, Jun Shao, Zhidan Li, Ran Zhou, Li Chen, Dan Zhang, Yuancun Zhao, Wei Liu, Yupeng Li, Ping He, Jing-wen Lin, Yuan Wang, Kang Zhang, Lu Chen, Weimin Li

**Affiliations:** 1 Department of Respiratory and Critical Care Medicine, National Clinical Research Center for Geriatrics, Center of Precision Medicine, Precision Medicine Key Laboratory of Sichuan Province, Frontiers Science Center for Disease-related Molecular Network, West China Hospital, West China School of Medicine, Sichuan University, Chengdu, Sichuan Province China; 2 Key Laboratory of Birth Defects and Related Diseases of Women and Children, West China Second University Hospital, State Key Laboratory of Biotherapy, Sichuan University, Chengdu, China; 3 Department of Neurology, State Key Laboratory of Biotherapy and Cancer Center, West China Hospital, Sichuan University, Chengdu, China; 4 Center for Biomedicine and Innovations, Faculty of Medicine, Macau University of Science and Technology and University Hospital, Macau, China

**Keywords:** Lung cancer, Bioinformatics

## Abstract

Lung adenocarcinoma (LUAD) and squamous carcinoma (LUSC) are two major subtypes of non-small cell lung cancer with distinct pathologic features and treatment paradigms. The heterogeneity can be attributed to genetic, transcriptional, and epigenetic parameters. Here, we established a multi-omics atlas, integrating 52 single-cell RNA sequencing and 2342 public bulk RNA sequencing. We investigated their differences in genetic amplification, cellular compositions, and expression modules. We revealed that LUAD and LUSC contained amplifications occurring selectively in subclusters of AT2 and basal cells, and had distinct cellular composition modules associated with poor survival of lung cancer. Malignant and stage-specific gene analyses further uncovered critical transcription factors and genes in tumor progression. Moreover, we identified subclusters with proliferating and differentiating properties in AT2 and basal cells. Overexpression assays of ten genes, including sub-cluster markers *AQP5* and *KPNA2*, further indicated their functional roles, providing potential targets for early diagnosis and treatment in lung cancer.

## Introduction

Lung cancer remains the leading cause of malignant tumor-related mortality worldwide, its etiological and biological heterogeneity contributes significantly to therapeutic failure and further unfavorable survival outcomes.^1^ Lung cancer can be classified into non-small cell lung cancer (NSCLC) and small cell lung cancer (SCLC). NSCLC, accounting for 85% of the lung cancer cases, predominantly consists of lung adenocarcinoma (LUAD) and lung squamous carcinoma (LUSC), two subtypes defined according to an integration of clinical and histologic features of tumors.^2,3^

Genetic, epigenetic, and microenvironmental characteristics could influence cellular programs and lead to disparate disease pathogeneses of NSCLC. Uniform therapeutic strategies might fail due to the underlying heterogeneity, resulting in a poor prognosis. There is a dichotomy between the therapeutic response in LUAD and LUSC patients, indicating distinct cellular compositions in these two subtypes of tumors. However, the differences in heterogeneity and cellular compositions between LUAD and LUSC and to what extent these cellular compositions impact patients’ survival remains largely unexplored. Therefore, a better understanding of the various sources of heterogeneity in lung cancer, regarding genetic, epigenetic, cellular, and microenvironmental characteristics, is a critical goal with broad implications for therapy.

Single-cell RNA sequencing (scRNA-seq) has emerged as a powerful method to comprehensively explore the cell-type composition of NSCLC resection specimens, thereby overcoming the technical barriers that have hampered understanding of intro-NSCLC heterogeneity. Previous studies exploring lung cancer at the single-cell level have primarily focused on immune and infiltrating cell populations^4^ or have specifically targeted a subset of readily identifiable cell types.^5^ A recent study used bulk RNA sequencing to characterize 305 East Asian LUAD patients genetically and transcriptionally,^6^ but did not characterize tumor cell types due to a lack of information on cell composition at single-cell resolution, leaving open the question of what cell types comprise the tumor bulk. While scRNA-seq can address those challenges, it is hindered by limited sample size and high financial cost. Combining the scRNA-seq results of a limited number of representative tumors with existing bulk data from large cohorts can help decipher the differences between two major subtypes of NSCLC.

Here, we used an integrative approach to understand NSCLC heterogeneity, combining scRNA-seq from patients with LUAD and LUSC at multiple tumor stages, as well as public bulk RNA-seq, whole-genome sequencing (WGS), and ATAC-seq datasets. Our scRNA-seq identified various cell types known to be present in lung cancer, including type II alveolar cells (AT2), basal cells, and a variety of myeloid and lymphoid cells. We revealed distinct cellular compositions in major lung cancer subtypes and investigated identified poor-prognosis-related subgroups. Gene module analysis and overexpression experiments revealed several important genes that may play functional roles in the early stage of tumor progression or subclusters of AT2 and basal cells, paving the way for potential early-stage interventions against lung cancer.

## Results

### Constructing a multi-omics atlas for LUAD and LUSC

**Fig. 1:**
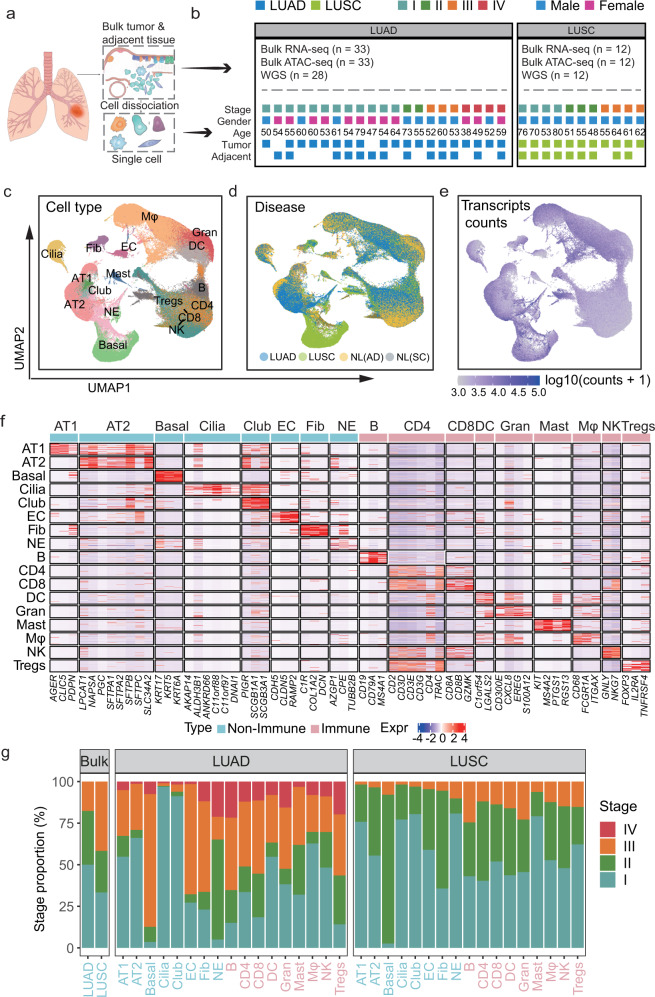
High-resolution cell-type mapping of LUAD and LUSC samples in both tumor and adjacent non-malignant tissues. **a** Workflow of the experimental design and analysis. **b** Summary of samples and patient clinical characteristics: the number of bulk RNA-seq, ATAC-seq, and WGS of WCH data (upper); the tumor stage, gender, age, and surgery site of patients with single-cell sequencing (Bottom). **c**–**e** UMAP of the 293,432 cells profiled here, with each cell color-coded for (left to right): cell types (**c**), the sample type of origin (tumor or adjacent tissues) (**d**), and the transcript expressed level (**e**). **f** Heatmap for visualization of the single-cell expression pattern of cell-type-specific gene markers. Immune cells were labeled with pink, non-immune cells were labeled with blue. **g** Percentage of samples and cells from four clinical stages for 17 types of cells and bulk RNA-seq

To comprehensively interrogate the cell-type composition and transcriptional profiles of NSCLC, we generated a single-cell and multi-omics atlas for LUAD and LUSC. In total, 52 fresh surgical resections of lung tumor and adjacent tissues were obtained from 32 untreated patients at West China Hospital (WCH), including 11 paired primary and adjacent LUAD samples, 8 unpaired LUAD and 2 adjacent samples, and 9 paired primary and adjacent LUSC patients and 2 additional primary LUSC samples, in a total of 21 lung adenocarcinoma and 11 squamous carcinoma patients (Fig. 1a, b, Supplementary Fig. 1a, and Supplementary Table 1). To perform scRNA-seq, cells were dissociated, sorted for viability, and profiled using 10X Genomics protocol. A total of 220,716 cells passed the quality control, with 127,593 cells originated from tumor tissues, and 93,123 from corresponding adjacent lung tissues. In all, 17,277 genes were detected with 1424 genes per cell on average, highlighting the high quality of our dataset. We also integrated our previous datasets including whole-genome sequencing (WGS), ATAC-Seq, and bulk RNA sequencing from an independent cohort of 45 lung cancer patients (Fig. 1b) and 4 benign lung tumor patients.^7^ Taken together, we constructed a multi-omics atlas for LUAD and LUSC at both bulk and single-cell resolution.

To define each cell type, we used Seurat^8^ and Harmony^9^ to process a total of 293,432 cells from ours and a previous study^5^ for quality control, normalization, batch effect correction, and clustering. All cells were visualized by Uniform Manifold Approximation and Projection (UMAP)^10^ and clustered to specific cell types based on the expression levels of signature genes with known populations^11^ and every sample showed differences in cell composition (Supplementary Fig. 1b). We identified 17 major cell populations across tumor and adjacent tissues in LUAD and LUSC (Fig. 1c, d and Supplementary Table 1), with an average of 5050 unique transcripts per cell type (Fig. 1e). All cell types were further validated and refined by SingleR^12^ (Supplementary Fig. 1c, d) and AUCell^13^ (Supplementary Fig. 1e). The classified cell populations could be divided into two groups, the non-immune cells, including basal cells (Basal), alveolar type II cells (AT2), alveolar type I cells (AT1), ciliated cells (Cilia), club cells (Club), endothelial cells (EC), fibroblasts (Fib), and neuroendocrine cells (NE). The remaining cells were nine immune cell populations comprising the tumor immune microenvironment, including B cells (B), CD4 + T cells (CD4), CD8 + T cells (CD8), dendritic cells (DC), granulocytes (Gran), mast cells (Mast), macrophages (Mφ), natural killer cells (NK), and regular T cells (Tregs) (Fig. 1f). Finally, we assigned each cell population to a tumor stage based on its originated patient categorized by the 8th edition of TNM classification of lung cancer.^14^ Cells from the early stage (stages I and II) accounted for 63.2% of the cells in LUAD, whereas ~87% of LUSC cells came from early-stage samples (Fig. 1g). Together, our scRNA-seq analyses uncovered multiple immune and non-immune cell types in both LUAD and LUSC, revealing intratumor heterogeneity and the tumor microenvironment in different subtypes of lung cancer.

### Charting malignant cells heterogeneity in NSCLC

**Fig. 2:**
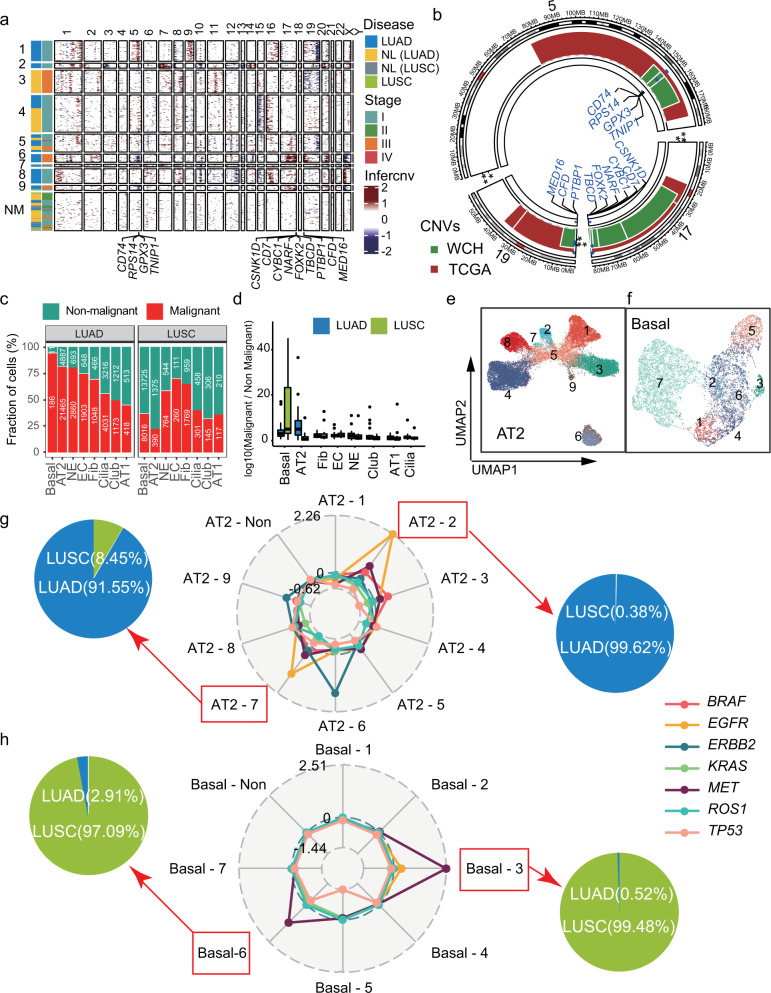
Identification of malignant cells and subclones based on the inferred CNVs. **a** Identification of genetic subclones based on inferred amplification or deletions of specific chromosome regions from scRNA-seq. The non-malignant (NM) cells showed no obvious CNVs. Thirteen genes with significant amplification were labeled. **b** Circus plot of CNVs on chromosomes 5, 17, 19 in WCH and TCGA. Thirteen genes identified from scRNA-seq were validated by whole-genome sequencing (WGS) data from both WCH (green) and TCGA (red) cohorts. **c** Percentage of malignant (red) and non-malignant (green) cells in eight non-immune cell types. **d** Comparison of the ratio between the number of malignant and non-malignant cells in LUAD (blue) and LUSC (green). **e**, **f** UMAPs of malignant AT2 (**e**) and basal cells (**f**) based on inferred CNVs. **g**, **h** The radar plot showed the average inferred-CNV score of driver genes in each subtype of AT2 (**g**) and basal (**h**). Pie charts presented the percentage of cells from LUSC or LUAD

To interrogate both inter- and intratumoral heterogeneity on malignant cells of NSCLC, we first classified cells into malignant and non-malignant cell types (Fig. 2a). For each cell type, we inferred copy-number variants (CNV) based on the average expression of 100 genes in each chromosomal region using inferCNV.^15–19^ For example, we identified large-scale amplifications in AT2 cells of LUAD patients, such as *CD74*, *RPS14*, *GPX3*, *TNIP1* of chr5, *CSNK1D*, *CD7*, *CYBC1*, *NARF*, *FPXK2*, *TBCD* of chr17, and *MED16*, *CFD*, *PTBP1* of chr19 (Fig. 2b), consistent with the hallmarks from WGS of WCH^7^ and TCGA datasets.^20^

To explore the propensity of malignant cells in all non-immune cell types, we compared the ratio of malignant cells to non-malignant cells. The dominant malignant cells in LUAD and LUSC were AT2 and basal cells, respectively (Fig. 2c, d), suggesting the importance of AT2 and basal cells in the formation of heterogeneity of lung cancer. According to the inferred CNVs, we divided each cell type into subclones (Fig. 2e, f). To further identify the key driver genes and potential therapeutic targets of each sub-clone, we calculated the amplification of the genes with critical clinical values, including *EGFR, KRAS, BRAF, ERBB2*, and *MET* (Supplementary Fig. 2a and Supplementary Table 2). For example, we revealed the peaked amplification of *EGFR* in subclones 2 and 7 of AT2 (Fig. 2g) and sub-clone 1 of AT1 cells, sub-clone 5 of NE cells (Supplementary Fig. 2a). The majority of the cells from these subclones are from LUAD patients, consistent with the notion that AT1 could be renewed by mature AT2 cells through the EGFR-KRAS pathway^21^ (Fig. 2g). Furthermore, targeted therapies have shown better progression-free survival (PFS) and higher objective response rate (ORR) compared with conventional cytotoxic therapy, and *EGFR* amplifications were found to be related to the sensitivity to TKI therapies.^2^ We also found that *MET* amplification was enriched in subclones 3 and 6 of basal cells, which were mainly from LUSC patients (Fig. 2h), in line with the role of *MET* as a prognostic of LUSC could promote a progressive tumor phenotype.^22^ Taken together, our results suggested that specific subclones of AT2 and basal cells might be the potential therapeutic targets of *EGFR* and *MET* in lung cancers, which could further enhance our understanding of the different responses in clinical therapy.

### Identifying recurrent cellular composition modules

**Fig. 3:**
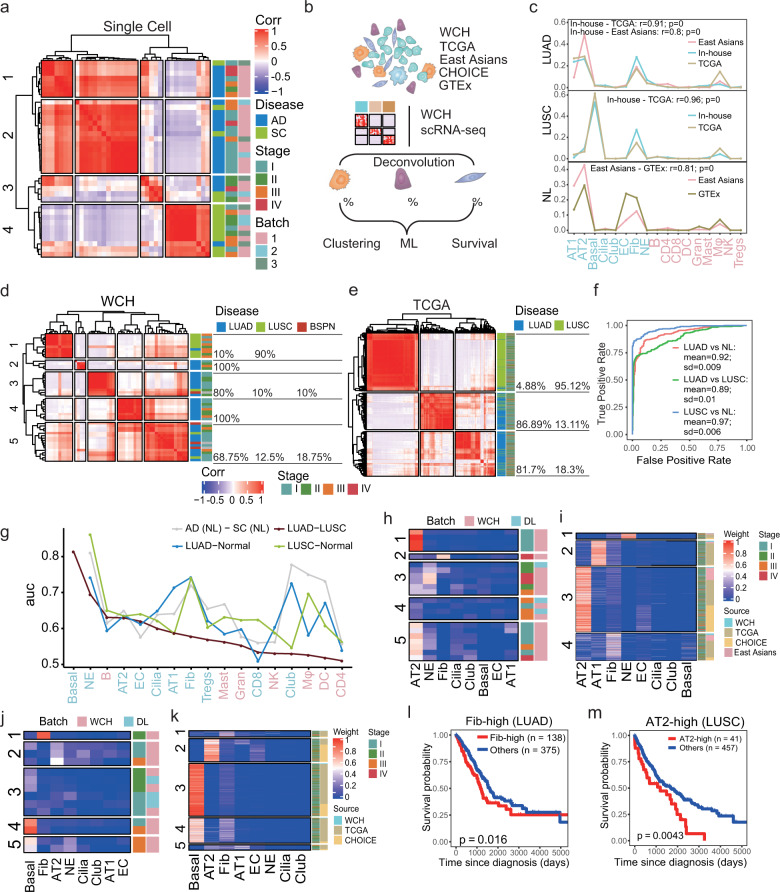
Distinct cellular composition modules of LUAD and LUSC. **a** Pearson correlation between cell compositions of malignant cells in scRNA-seq. **b** Flowchart overview of the deconvolution workflow in which our scRNA-seq was used to provide cell-type-specific genes. **c** Line plots show the cell composition of 17 cells deconvoluted from different sources of bulk RNA-seq. The correlations were calculated using Pearson correlation. **d**, **e** The Pearson correlation between cell weights of samples from WCH (**d**) and TCGA (**e**). LUAD and LUSC tended to be clustered together. The proportions of different disease types are labeled on the right. **f** ROC was performed for cell weights of different lung cancer subtypes and normal lung tissue from independent cohorts. Multiple cross-validations were performed to generate reliable values for different groups. The mean area under the curve (AUC) of LUAD vs normal tissue was 0.92 (SD = 0.009), LUSC vs normal tissue was 0.97 (SD = 0.006); LUAD vs LUSC was 0.89 (SD = 0.01). **g** Prioritizing the most affected cell types in LUAD and LUSC progression by ranking the AUC scores derived from the Augur algorithm. **h** Heatmap of the proportion of non-immune cells in patients from LUAD. **i** Heatmap of the deconvoluted weights of non-immune cells based on bulk RNA-seq data from LUAD patients. Patients could be separated into four groups, including NE-high (1), AT1-high (2), AT2-high (3), and Fib-high (4). **j** Heatmap of the proportion of non-immune cells in patients from LUSC. **k** Heatmap of the deconvoluted weights of non-immune cells based on bulk RNA-seq data from LUSC patients. Five groups, including Fib-high (1), AT2-high (2), Basal-high (3), Basal-Fib hybrid (4), and Hybrid (5). **l** Kaplan–Meier survival curves for patients with LUAD (*n* = 513), stratified for the Fib-high group and the rest. *P* value was calculated using the log-rank test. **m** Kaplan–Meier survival curves for patients with LUSC (*n* = 498), stratified for the AT2-high group and the rest. *P* value was calculated using the log-rank test

To comprehensively represent the intratumoral heterogeneity among the malignant cells, we identified cellular compositions in scRNA-seq and then sought to discover the recurrent cellular composition modules in large cohorts from bulk RNA-seq. We first hierarchically clustered the samples based on the compositions of all non-immune cells in LUAD and LUSC in scRNA-seq. Notably, LUAD and LUSC tended to cluster together, indicating that these two subtypes had distinct cellular compositions (Fig. 3a).

To validate this observation, we sought to assess the cell compositions from large cohorts with tumorous and normal lung bulk RNA-seq, including TCGA (*n* = 1116), WCH RNA-seq (*n* = 49), East Asians group (*n* = 260), CHOICE (*n* = 490), and GTEx (*n* = 427) (Supplementary Table 3). Since the bulk samples consisted of a heterogeneous mixture of various cell types, we developed a deconvolution workflow inferCC (infer cellular composition based on scRNA-seq expression signatures) to evaluate the relative fractions of diverse cell types (Fig. 3b, see “Methods”). We found that the cell compositions were highly consistent despite different sources of bulk RNA-seq in LUAD, LUSC, and normal lung (Pearson correlation *r* > 0.8, Fig. 3c). Indeed, the cell composition could distinguish the LUAD and LUSC in WCH (Fig. 3d), TCGA (Fig. 3e), and CHOICE (Supplementary Fig. 2b), and the LUAD and adjacent tissue in an East Asian cohort (Supplementary Fig. 2c). We next confirmed the reliability by the area under the curve (AUC) analysis of our deconvolution method. Based on these cell compositions, we were able to correctly classify the lung cancer subtypes with high accuracy using a support vector machine (SVM) (Fig. 3f, AUC = 0.89, SD = 0.01), suggesting the cellular compositions deconvoluted by inferCC could be used to classify the lung cancer patients. Taken together, our analyses revealed that LUAD and LUSC harbored distinct cellular compositions, which played important roles in forming their heterogeneity.

To prioritize the cell types most responsive to biological perturbations in our scRNA-seq data, we performed Augur^23^ among LUAD, LUSC tumor tissues, and corresponding adjacent samples. Notably, the non-immune cells showed higher rankings (using AUC as proxy) than immune cells when comparing LUAD to LUSC. We further identified that basal is one of the main sources of heterogeneity between LUAD and LUSC, whereas fibroblast and NE are the key cell types that distinguish two tumor subtypes from their adjacent tissues, indicating that these cell types are of importance in NSCLC (Fig. 3g). Next, we sought to identify subgroups of LUAD and LUSC based on their cellular compositions. In LUAD, we identified four groups: AT2-high, Fib-high, AT1-high, and NE-high (Fig. 3h) in our scRNA-seq. These four subtypes were further confirmed using the TCGA cohort (Fig. 3i). Notably, the patients classified as Fib-high were related to poor prognosis in LUAD in TCGA (*P* = 0.016, log-rank test, Fig. 3l), while other groups were not related to the survival (Supplementary Fig. 2d–f). In LUSC, we discovered four main groups: basal-high, fib-high, AT2-high, and NE-high (Fig. 3j) in our scRNA-seq, which were validated using the TCGA cohort (Fig. 3k). Importantly, we found that the AT2-high (*P* = 0.0043, log-rank test, Fig. 3m), basal-high (*P* = 0.0038, log-rank test, Supplementary Fig. 2g) and low compositions of basal-fib (*P* = 0.027, log-rank test, Supplementary Fig. 2h), could contribute to poor survival of LUSC, whereas the Fib-high (Supplementary Fig. 2i) and hybrid (Supplementary Fig. 2j) was not related to the survival. Therefore, we developed a deconvolution method that could be applied to patient classification for bulk RNA-seq, and our results indicated that specific cellular compositions may have important potential values in predicting the prognosis of lung cancer.

### Modified cell–cell interactions of LUAD and LUSC

**Fig. 4:**
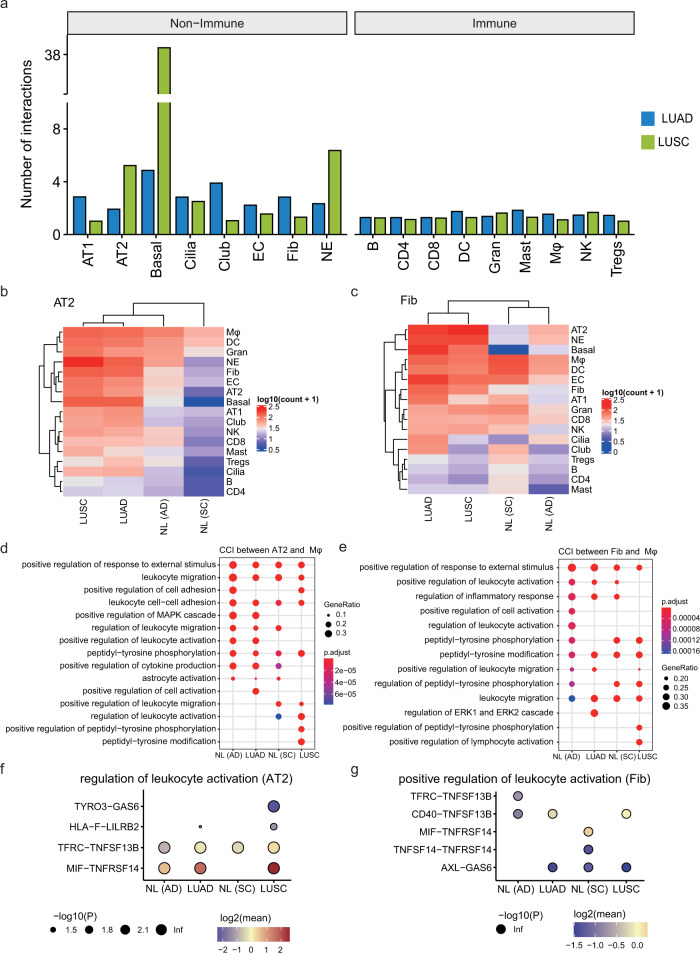
The interaction between non-immune and immune cells in LUAD and LUSC. **a** The bar plot showed the number of interactions of each cell type in LUAD (green) and LUSC (blue). **b** Heatmap showed the number of interactions between AT2 and other cells in LUAD, LUSC, and its corresponding adjacent samples. **c** Heatmap showed the number of interactions between fibroblast and other cells in LUAD, LUSC, and its corresponding adjacent samples. **d** The GO annotation of ligand–receptor pairs between AT2 and Mφ. The dot size represented the number of genes. The color scale represented the adjusted *P* value. **e** The GO annotation of ligand–receptor pairs between fibroblast and Mφ. The dot size represented the number of genes. The color scale represented the adjusted *P* value. **f** Overview of selected ligand–receptor interactions between AT2 and Mφ enriched in regulation of leukocyte activation pathway; *P* values are indicated by circle size; scale is shown below the plot. The means of the average expression level of interacting were indicated by color. **g** Overview of selected ligand–receptor interactions between fibroblast and Mφ enriched in regulation of leukocyte activation pathway; *P* values were indicated by circle size; scale was shown below the plot. The means of the average expression level of interacting were indicated by the color

Altered intercellular interaction plays an important role in tumor progression. To elucidate the redistribution of each kind of ligand–receptor interactions in two subtypes of NSCLC, we performed cell–cell interaction (CCI) analysis and calculated the numbers of receptor–ligand parings in each cell type based on the CellPhoneDB.^24^ The intercellular interactions of non-immune cell types including AT2 and Fib cells were shifted between LUAD and LUSC (Fig. 4a). In contrast, cellular interactions of immune cells showed no obvious variation between two subtypes. Our results highlighted the difference of cell–cell interactions between immune and non-immune cells, and two subtypes of NSCLC.

Given the Fib-high in LUAD, AT2-high, and Basal-Fib hybrid in LUSC were related to poor prognosis, we next focused on AT2 and fibroblasts in terms of their CCI of LUAD and LUSC. Our results showed that distinct increases CCI in both LUSC and LUAD comparing to their adjacent tissues (Fig. 4b, c). Furthermore, the GO annotation indicated that the ligand and receptors between AT2, Fib, and Mφ were enriched in leukocyte migration, activation, and peptidyl-tyrosine-related pathways (Fig. 4d, e). For instance, we identified the receptor–ligand parings between tyrosine kinase receptors *TYRO3* and *GAS6*, the immunosuppressive receptor *LILRB2* and *HLA-F*, were specifically occurred in the AT2 cells of LUSC (Fig. 4f), whereas the interaction between *AXL* and *GAS6* was observed in fibroblasts in LUAD but not in their adjacent tissues (Fig. 4g), consistent with their potential functions in NSCLC. Indeed, a previous study has revealed that the *TYRO3* and *GAS6* may promote the Mφ polarization to the tumor-promoting M2 phenotype,^25^ while *LILRB2* was also related to the in NSCLC can promote the polarization of tumor-infiltrating myeloid cells to the inflammatory phenotype.^26^ Our results revealed the increased CCI in both LUAD and LUSC and some receptor–ligand parings may play functional roles in NSCLC.

### Malignant- and stage-specific gene alterations

**Fig. 5:**
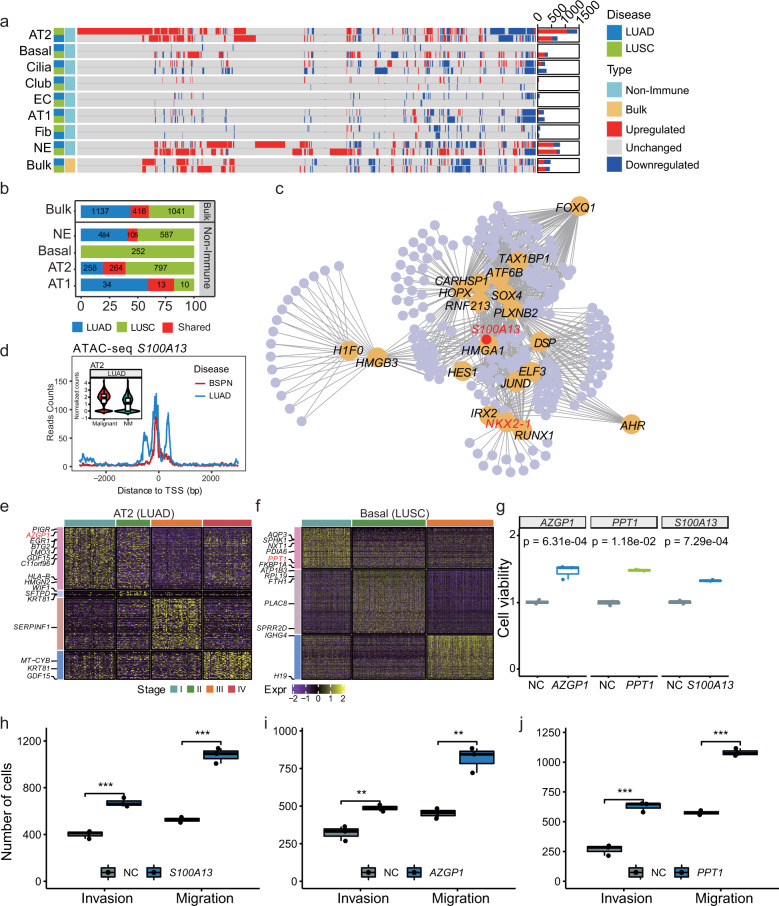
Distinct gene expression patterns of non-immune cells in LUAD and LUSC. **a** Distribution of DEGs in each cell type and bulk RNA-seq across the two lung cancer subtypes. Bar plots showed the number of genes. Each row represented one cell type in a specific lung cancer subtype, and each column represented one gene. Red, upregulated (average logFC >0.25 for scRNA-seq and logFC >1 for bulk RNA-seq, adjusted *P* value < 0.05); blue, downregulated (average logFC < -0.25 for scRNA-seq and logFC < -1 for bulk RNA-seq, adjusted *P* value <0.05); gray, unchanged (|average logFC | < 0.25 for scRNA-seq and |logFC | <1 for bulk RNA-seq). **b** Bar plots showed the percentage of upregulated genes in malignant cells that were LUAD-specific (blue), LUSC-specific (green) and shared (red), respectively. **c** The regulatory networks of upregulated DEGs of AT2 from LUAD. Only the top regulators identified by LeMoNe were drawn. **d** The line plot showed the number of reads covered of ATAC-seq region around 3000 bp up- and down-stream of *S100A13* TSS each. The violin plot of normalized gene expression in malignant and non-malignant AT2 cells from LUAD was placed in the left top. **e**, **f** Heatmaps of the expression of tumor-stage-specific modules of AT2 from LUAD (**e**) and basal cells from LUSC (**f**). **g** The cell viability of *AZGP1*, *S100A13*, and *PPT1* overexpression H1299 cells. Cell viability detection was completed by CCK8 detection. The *P* value was calculated using a *t* test. **h**–**j** The boxplot showed the number of invasion and migration cells of *S100A13* (**h**), *AZGP1* (**i**), and *PPT1* (**j**) overexpression H1299 cells. The *P* value was calculated by Student’s *t* test

To unveil the association between gene expression variations and intra/inter-tumor heterogeneity, we performed differential expression analysis between malignant and non-malignant cells for each cell type in LUAD and LUSC. Compared with non-malignant cells, thousands of differentially expressed genes (DEGs) were identified in malignant cells. Notably, AT2, basal, NE cells harbored the highest numbers of up or downregulated genes in the malignant cells (Fig. 5a and Supplementary Tables 4 and 5). To identify the common upregulated DEGs shared between two subtypes, we compared the percent of subtype-specific and common DEGs in different cell types. For instance, DEGs of AT2 and NE cells had only 20% and 8.93% common genes between LUAD and LUSC (Fig. 5b). We further used the cell specificity index tau^27^ and revealed that the upregulated DEGs of non-immune cells had high cell-type specificity (mean tau = 0.76 in LUAD, and 0.73 in LUSC). These results indicated that the non-immune cells were divergent between LUAD and LUSC and the DEGs were highly cell-type specific.

To better understand the molecular mechanism of AT2 and basal underlying the pathogenesis of lung cancer, we constructed a TF regulatory network based on upregulated genes. The lineage-specific tumor suppressor *NKX2-1*^28^ was identified as one of the key regulators of AT2 cells in LUAD (Fig. 5c and Supplementary Table 6), consistent with its amplification in 10% of LUAD.^29^ Our network revealed that *S100A13*, a gene involved in calcium-binding and cell cycle progression,^30^ is regulated by *NKX2-1* (Bayesian score = 5.76, Supplementary Table 6). Indeed, *S100A13* had higher ATAC-seq peaks around the transcription start site (TSS) in LUAD compared to benign solitary pulmonary nodules (BSPN), consistent with its higher expression in malignant AT2 (Fig. 5d). The network analysis in LUSC identified key TFs including *KLF5* and *MYC* in basal cells (Supplementary Fig. 3a), consistent with its amplification and overexpression in lung cancer patients, acting as the prognostic markers of early-stage tumors.^29,31^

Next, we performed the Gene Ontology (GO) and Disease Ontology Semantic and Enrichment (DOSE) analyses for upregulated genes of malignant cells. In LUAD, AT2 cells were enriched with genes from responses of unfolded protein, immune cells, and regulation of mRNA metabolic process (Supplementary Fig. 3b). In LUSC, the basal cells were enriched with immune response, protein refolding, type I interferon, leukocyte, and cell activation (Supplementary Fig. 3c). Interestingly, we observed that a common enriched pathway of AT2, club, NE cells, and bulk RNA-seq was the non-small cell lung carcinoma in LUAD (Supplementary Fig. 3d), suggesting that the high relevance of multiple cell types in non-small cell lung cancer. Notably, compared to bulk RNA-seq, scRNA-seq identified more cell-type-specific genes enriched in non-small cell lung carcinoma pathways in both LUAD (Supplementary Fig. 3e) and LUSC (Supplementary Fig. 3f), highlighting the potential cell-type/stage-specific effects. Our results demonstrated that in-depth interpretation of gene expression data from scRNA-seq comparison could provide a more comprehensive understanding of the transcriptional diversification in LUAD and LUSC.

To investigate the tumor-stage-specific regulation, we performed the differential gene expression analysis (DEG) for each cell type. AT2, basal, NE and fibroblast cells had higher numbers of DEGs comparing to other non-immune cells (Supplementary Fig. 3g and Supplementary Table 7), consistent with the pattern of that higher number of non-immune malignant cells. We further constructed gene modules in AT2 (LUAD) and basal (LUSC) cells, which revealed that many tumor-related genes in cells from stage-I patients, including *PIGR, AZGP1, BTG2, EGR1*, and *LMO3* in AT2 cells from LUAD (Fig. 5e), and *AQP3, SPHK1, PPT1*, and *PDIA6* were found in basal cells from LUSC (Fig. 5f).

Finally, we explored the potential functions of the upregulated gene *S100A13* in malignant cells and stage-I upregulated gene *AZGP1* of AT2 in LUAD and *PPT1* of basal cells in LUSC.

*S100A13* is a member of S100 calcium-binding protein, functioning in cell cycle progression and membrane permeability.^30^ *AZGP1* plays an important role in lipid mobilization and contributes to malignancy-related cachexia and *PPT1* encodes a small glycoprotein involved in lipid catabolism by removing thioester-linked fatty acyl groups from cysteine residues. We first validated their protein expression levels by using immunofluorescence staining of lung tumors and normal tissues (Supplementary Fig. 4a, c, e). Furthermore, overexpression of *S100A13* (*P* = 7.29e-4, *t* test), *AZGP1* (*P* = 6.31e-4, *t* test), and *PPT1* (*P* = 1.18e-4, *t* test), significantly increased the proliferation of lung cancer H1299 cells (Fig. 5g). In addition, transwell assays also confirmed that *AZGP1*, *S100A13*, and *PPT1* enhanced migration and invasion capacity of H1299 cells (Fig. 5h–j and Supplementary Fig. 4b, d, f), suggesting the oncogenic role of these genes. Taken together, our results identified transcriptomic alterations in LUAD and LUSC, and revealed the potential functions of *AZGP1, S100A13*, and *PPT1* genes in tumorigenesis that were identified in early-stage DEGs, indicating them as potential biomarkers and targets for early diagnosis and therapy.

### Distinct characteristics of AT2 subclusters in LUAD

**Fig. 6:**
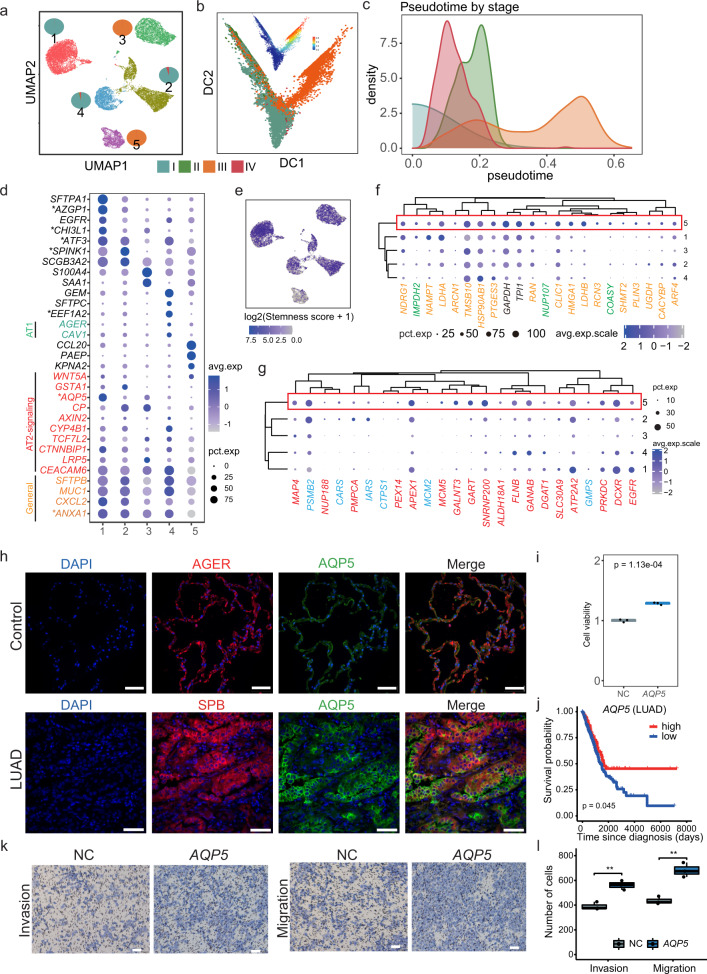
Subclusters and pseudotime analysis of the malignant AT2 cells in LUAD. **a** UMAP showed subclusters of 21,465 malignant AT2 cells in LUAD, with pie charts illustrated the fraction of each stage in each sub-cluster. **b** Pseudotime analysis using diffusion map of malignant AT2, colored with different stages. The diffusion map colored with pseudotime was plotted on the top. **c** Density of the four stages in the inferred pseudotime score. Stages I, II, and III showed a relatively distinct pattern, while stage IV that with fewer patients was placed between stages I and II. **d** Dot plot showed the average expression level (the intensity of blue) and percentage of expressed cells (the dot size). Expression of sub-cluster makers and indicated AT2, and AT2 cell markers and percent of cells in population with detected expression (dot size). General markers of AT2 (yellow), markers of AT1 (green), and markers of AT2-signaling selective (red) were colored. **e** UMAP plot showed the log2 transformed stemness score calculated using the mean level of expression of AT2-signaling markers. The sub-cluster 1, 2, and 3, where AT2-signaling-like markers were highly expressed, showed a higher stemness score. **f** The expression status of potential drug targets and potential prognostic biomarkers of LUAD. The drug targets were labeled in green, prognostic biomarkers colored in yellow, and the common markers from both sets were colored in black. **g** The expression patterns of targetable mutations (red) and potential drug targets of *EGFR* mutant (blue) of LUAD. **h** The expression of *AQP5* in LUAD. Immunofluorescence staining indicated the location of *AQP5* level in lung cancer cells (green), SPB (Surfactant protein B) was the marker of lung adenocarcinoma cells (red); AGER (advanced glycosylation end-product specific receptor) was the marker of AT1 cells in normal lung, the cell nucleus was co-stained with DAPI (blue); Scale bar, 50 μm. **i** The cell viability of H1299 cells after overexpression of *AQP5*. Cell viability detection was completed by CCK8 detection. The *P* value was calculated using *t* test. **j** Kaplan–Meier survival curves for patients with LUAD (*n* = 513), stratified for patients highly expressed *AQP5* and rest patients. *P* value was calculated using the log-rank test. **k** The invasion and migration of *AQP5* overexpression H1299 cells. Transwell assays were conducted for cell migration (without matrigel) and invasion abilities (with matrigel). Scale bar, 100 μm. **l** The boxplot shows the number of invasion and migration cells of *AQP5* overexpression H1299 cells. The *P* value was calculated by Student’s *t* test

To further understand the subclusters of the critical AT2 cells in LUAD, we focused on the 21,465 malignant AT2 cells and their five subclusters. Interestingly, these clusters tended to be stage-specific. Clusters 1, 2, and 4 included cells mostly from patients with early-stage (stage I and II) lung cancer, whereas clusters 3 and 5 were dominated by cells from advanced-stage (stage III and IV) patients (Fig. 6a). To illustrate the transcriptional trajectory in AT2 cells, we performed a pseudotime analysis using URD.^32^ Notably, the inferred pseudotime results reflected stage from I to II, and then to III, with 280 cells from stage IV in between (Fig. 6b, c). These results were in line with the stage-specific gene modules (Fig. 5e), suggesting a transcriptomic divergence of AT2 cells between early- and advanced-stage patients.

To assess the biological function of each sub-cluster, we compared the marker genes of each cluster to those from a single-cell atlas of the human lung,^11^ in which genes were classified into the AT2 general and AT2-signaling selective (AT2-s), AT1 general marker genes, and sub-cluster-specific genes. Interestingly, we observed some concordant expression patterns between the normal and malignant AT2 cells. The AT2 general markers showed broad expression in all clusters, while other clusters exhibited a unique repertoire of cluster-specific markers (Fig. 6d and Supplementary Fig. 5a, and Supplementary Table 8). For instance, cluster 4 had the highest expression of *AGER*^33^ and *CAV1*,^34^ which are the AT1 markers, suggesting that these cells may undergo a transition from AT2 to AT1 cells. Notably, AT2-signaling (AT2-s) genes, including a ligand of WNT pathway (*WNT5A*), regulatory protein (*CTNNBIP1*) co-receptor (*LRP5*) and transcription factor (*TCF7L2*), was reported to be homologous to the rare subpopulation of WNT-active AT2 cells and might be alveolar stem cells.^11^ Clusters 1, 2, 3, and 4 tended to have high expression of AT2-signaling genes, while cluster 5 had the lowest expression of *AQP5*, *AXIN2,* and *GSTA1* (Fig. 6d). Indeed, when comparing the stemness scores among clusters, cluster 5 showed a relatively lower percentage of cells with stemness potential when comparing with other clusters (Fig. 6e), suggesting that cells from different tumor stages may have distinct compositions of cells with stemness potential.

Next, we compared the gene expression levels of prognostic biomarkers, drug targets (Fig. 6f), and targetable mutations (Fig. 6g) among these subclusters. They had relatively higher expression in cluster 5, indicating that our current prognostic markers and drugs were limited on genes from the differentiating cells from the advanced stage but not targeting the proliferating clusters at the early stage of the tumor.

To investigate the functional implications of the mark genes from proliferating clusters, we assessed seven genes, including general marker (*ANXA1*), cluster 1 (*AQP5, ATF3, AZGP1*, and *CHI3L1*), cluster 2 (*SPINK1*), and cluster 4 (*EFF1A2*). *ANXA1* was highly expressed in all clusters. We experimentally validated that ANXA1 protein was expressed in LUAD tumor and normal tissues by immunofluorescence staining (Supplementary Fig. 5b), and overexpression of *ANXA1* indicated it might promote tumor by enhancing proliferation (Supplementary Fig. 5c, *P* = 2.94e-3, *t* test), migration, and invasion ability (Supplementary Fig. 5d, e) in lung cancer H1299 cells, consistent with its function as an intercellular transport protein and interactor in cell division, migration and plasmin production.^35^ More importantly, for the cluster-specific genes, we revealed that immunofluorescence staining of *AQP5*, as well as the AT2 marker SPB, were upregulated in AT2 cells of LUAD tumors tissues compared with normal controls (Fig. 6h), and overexpression of *AQP5* in lung cancer H1299 cells enhanced proliferation (Fig. 6i, *P* = 1.13e-4, *t* test) and high level of *AQP5* was related to better prognosis (Fig. 6j, *P* = 0.045), and invasion ability of lung cancer cells, suggesting a tumor-promotion role of *AQP5* (Fig. 6k, l), in line with its role of promoting tumorigenesis by inducing the epithelial–mesenchymal transition (EMT) process.^36^ Similarly, other markers of cluster 1 (*ATF3, AZGP1,* and *CHI3L1*), cluster 2 (*SPINK1*), and cluster 4 (*EFF1A2*) were revealed to promote tumorigenesis by overexpression experiments (Supplementary Fig. 5f–h and Supplementary Fig. 6a–l). In sum, we uncovered clusters in malignant AT2 cells with distinct features with some resembled proliferating cells (clusters 1-4) while other (cluster 5) tended to be differentiating from patients in stage III, and the experimentally validated six marker genes may be of potential clinical value.

### Divergent subclusters of basal cells in LUSC

**Fig. 7:**
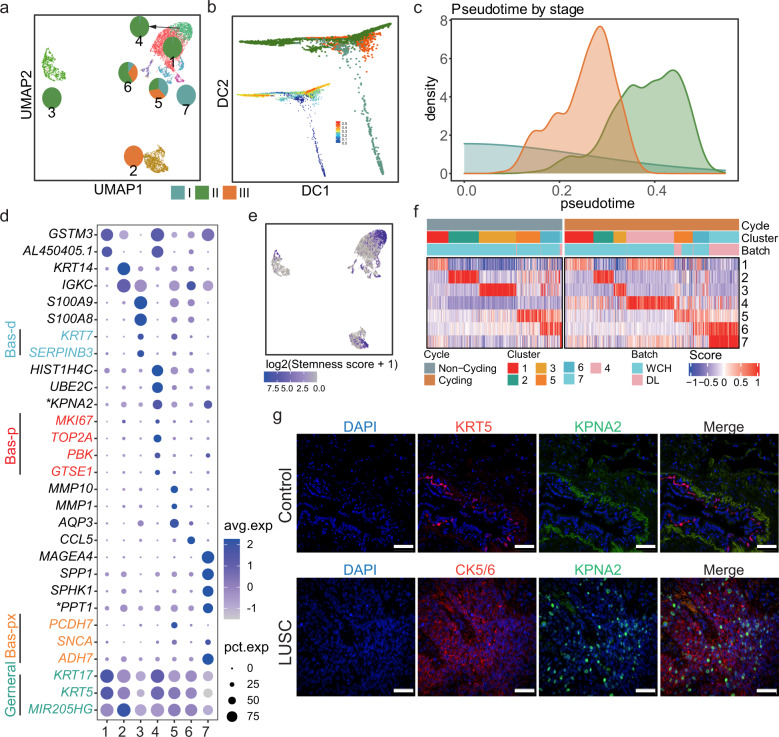
Subclusters and pseudotime analysis of the malignant basal cells in LUSC. **a** UMAP of 8016 malignant basal cells from LUSC, colored with different clusters, and the fraction of different stages of each cluster were labeled with a pie chart. **b** Diffusion map of malignant basal cells from LUSC, colored with different stages. The diffusion map colored with pseudotime was plotted at the left top corner. **c** Density of pseudotime colored with different stages. **d** Dot plot of the mean level of expression (dot intensity, blue scale) of cluster makers and indicated basal cell markers and percent of cells in population with detected expression (dot size). Generic markers colored in green, proliferation basal (Bas-p) markers colored in red, proximal basal (Bas-px) markers colored in yellow and differentiating basal (Bas-d) markers colored in blue. **e** UMAP plot showed the log2 transformed stemness score calculated using the mean level of expression of Bas-p markers. **f** Heatmap showed the cluster scores of all non-cycling cells (left) and cycling cells (right). Within each group, the cells were defined by maximal score, for cells mapping to one cluster. **g** The expression of *KPNA2* in LUSC. Immunofluorescence staining indicated the location of *KPNA2* level in lung cancer cells (green), CK5/6 (Cytokeratin 5/6) was used as the marker of lung squamous carcinoma cells (red), *KRT5* (Keratin 5) was used as the marker of basal cells in normal lung, the cell nucleus was co-stained with DAPI (blue); Scale bar, 50 μm

Basal cells were shown to be one of the key cell types in LUSC based on cell module analyses. To characterize the subclusters of basal cells, we focused on the 8202 malignant cells which can be divided into seven clusters. Clusters 1, 3, 4, and 7 predominantly consisted of basal cells from early-stage (stage I and II) patients, whereas clusters 5 and 6 were a mixture of different stages, and cluster 2 was all from stage III (Fig. 7a). In the pseudotime analysis, the cells from stage II were deviated from those from I and III (Fig. 7b, c), in line with stage II had the maximum number of stage-specific genes (Fig. 5f).

To understand the biological properties of each cluster, we compared our cluster-specific genes (Fig. 7d, Supplementary Fig. 7a, and Supplementary Table 8) to those from a human lung cell atlas.^11^ The basal general markers were broadly expressed in all clusters, while the markers of proximal basal (Bas-px) were highly expressed in cluster 7, and those of differentiating basal (Bas-d) were selectively expressed in cluster 3 (Fig. 7d). Notably, cluster 4 selectively expressed markers from proliferating basal (Bas-p), including *MKI67, TOP2A, PBK,* and *GTSE1*. We further utilized the stemness and cell cycling scores and revealed that cluster 4 had the highest stemness score and was mainly classified as cycling cells (Fig. 7e, f), indicating this cluster might have the characteristics of proliferating cells.

Finally, we experimentally investigated the cluster-specific *KPNA2* (karyopherin alpha 2) for cluster 4 and *PPT1* for cluster 7 (described in Fig. 5 as stage-I-specific gene). Increased expression of KPNA2 protein was observed in tumor tissues by immunofluorescence staining in comparison to normal lung tissues (Fig. 7g). Overexpression of *KPNA2* revealed its tumor-promotion role by increasing proliferation (*P* = 1.02e − 4, *t* test) (Supplementary Fig. 7b), migration and invasion of lung cancer H1299 cells (Supplementary Fig. 7c, d). Taken together, our results revealed clusters of malignant cells that resembled normal lung cell subclusters and a cluster (cluster 4) was with stem-like proliferating features in malignant basal cells, and its maker *KPNA2* might promote tumor proliferation in LUSC.

## Discussion

Inter and intratumor heterogeneity is a key factor contributing to poor prognosis and variation of therapy applications between LUAD and LUSC. The single-cell analysis represents a viable strategy to investigate heterogeneity and characterize recurrent cellular compositions, as well as tumor subtype- and stage-specific transcriptomic landscapes. However, extensive comparisons of heterogeneity in LUAD and LUSC are still limited, which is critical for precision therapy of lung cancer. Here, we performed scRNA-seq and multi-omics profiling of lung cancer from multiple LUAD and LUSC patients. To facilitate the community to use our dataset, we further built a website (http://lungcancer.chenlulab.com) providing interactive query gene expression, clustering of all the cell types and data downloads (Supplementary Fig. 7e).

CNVs were one of the sources in driving tumor progression and acquiring therapeutic resistance.^37^ Based on the inferred CNVs, malignant AT2 cells and basal cells from LUAD and LUSC could be divided into multiple subclones. Amplification of *EGF*R in LUAD and *MET* in LUSC were unevenly distributed in these subclones. While how the clinical implication of these copy-number alterations in malignant cells remained to be further explored, our single-cell CNVs profiles suggest that different subclones may have different responses to targeting treatment.

We further developed a deconvolution method for bulk RNA from large cohorts and found recurrent cellular compositions from both scRNA-seq and deconvoluted bulk RNA-seq. Importantly, we revealed that high percentages of Fib and AT2 contributed to poor survival of LUAD and LUSC, suggesting the potential roles of cellular compositions in predicting poor prognosis of lung cancer.

Our study included samples from tumor and adjacent tissues of patients from different tumor stages. Based on genes identified from the malignant-, stage-, and sub-cluster analyses, we experimentally demonstrated that some genes may play functional roles in tumor progression. Our results consistently highlighted the importance of AT2 cells in LUAD, and their subclusters showed proliferating and differentiating properties.AT2 cells, playing pivotal secretory and regenerative roles to maintain the homeostasis in the alveolus of the lung, are known to be a heterogeneous population and maybe the cells of origin of LUAD.^38^ Experimentally, this hypothesis is supported by immunofluorescence staining and overexpression experiments in H1299 cells, linking eight candidate genes (*AQP5*, *ATF3*, *AZGP1*, *ANXA2*, *CHI3L1*, *EEF1A2*, *SPINK1*, and *S100A13*) to their promoting roles in LUAD. Indeed, high expression of *AZGP1* was reported in multiple malignancy tumors such as lung cancer and breast cancer, which played a vital role in lipid mobilization and contributed to malignancy-associated cachexia.^39,40^ And *S100A13*, a member of the S100 family, was closely associated with aggressive invasive phenotype and angiogenesis.^30^ Functioning in delivering aminoacylate-tRNA to a site of the ribosome for decoding of mRNA, *EEF1A2* played an important role in translation and might also act as an oncogene in lung cancer and pancreatic cancer.^41,42^

In LUSC, some subclusters of malignant basal cells showed proliferating features. Their marker genes included *PPT1* and *KPNA2. PPT1* was reported as a molecular target of chloroquine derivates in melanoma cell lines, closely correlating with the poor prognosis in several cancers, such as breast cancer, clear cell renal cell carcinoma, head and neck squamous carcinoma, thyroid cancer, and colon adenocarcinoma.^43–45^ *KPNA2*, a nucleocytoplasmic transporter known to be involved in many cellular processes including differentiation, development, and immune response^46^ was also revealed to have the potential act as a stemness marker in LUSC. Overexpression of *KPNA2* was previously reported in LUAD tissues and negatively regulated by a novel transcription factor interferon regulatory factor-1 (IRF1).^47,48^ Our study revealed that *KPNA2* may be a proliferating marker and promote tumor progression in LUSC.

Notably, our study revealed distinct stemness-related subclusters in both LUAD (cluster 1 in AT2 cells) and LUSC (cluster 4 in basal cells). Cancer stem cells (CSCs) have been referred as “tumor-initiating cells” or “sphere-forming cells”, which promoted tumor progression by increasing tumor proliferation and angiogenesis.^49^ Multiple pathways, including Hedgehog, Notch, Nanog, Wnt and PI3K/AKT, are involved in CSCs-mediated tumor promotion.^50^ Thus, CSCs are considered as therapy target and yield an improved therapeutic window.^51^ Blocking CSCs-related signaling pathways provides promising strategy in tumor therapy, in a cohort of 40 glioblastoma patients who received vismodegib treatment (targeting Hedgehog pathway) for 7 days showed dramatically reduction of ex vivo CD133 + neutrosphere formation.^52^ In a 30 breast cancer patients with locally advanced and metastasis, Notch pathway inhibitor MK-0752 induced 11 patients had complete remission and 9 patients achieved stable disease.^53^ Besides signaling pathways, other CSCs-related targets involved surface marker,^54^ CSC microenvironment^55^ and aberrant metabolism.^56^ Our results revealed two stemness-like clusters in AT2 and basal cells, which could act as potential candidate targets in future precision lung cancer treatment.

In summary, our study integrated multi-omics analysis to establish a lung tumor atlas and extensively investigated the heterogeneity variation between LUAD and LUSC, which could be used as a valuable resource for understanding the unique mechanism of tumor progression in lung cancer subtypes, providing a blueprint for potential early-stage diagnosis and treatment for lung cancer.

## Supplementary information

supplementary materials

supplemental tables

## References

[1] Bray F (2018). Global cancer statistics 2018: GLOBOCAN estimates of incidence and mortality worldwide for 36 cancers in 185 countries. CA Cancer J. Clin..

[2] Herbst RS, Morgensztern D, Boshoff C (2018). The biology and management of non-small cell lung cancer. Nature.

[3] Shi JF (2019). Clinical characteristics and medical service utilization of lung cancer in China, 2005-2014: overall design and results from a multicenter retrospective epidemiologic survey. Lung Cancer.

[4] Guo X (2018). Global characterization of T cells in non-small-cell lung cancer by single-cell sequencing. Nat. Med..

[5] Lambrechts D (2018). Phenotype molding of stromal cells in the lung tumor microenvironment. Nat. Med..

[6] Chen, J. et al. Genomic landscape of lung adenocarcinoma in East Asians. *Nat. Genet.* **52**, 177–186 (2020). 10.1038/s41588-019-0569-6 32015526

[7] Wang, Z. et al. The open chromatin landscape of non-small cell lung carcinoma. *Cancer Res.* **79**, 4840–4854 (2019). 10.1158/0008-5472.CAN-18-3663 31209061

[8] Butler A, Hoffman P, Smibert P, Papalexi E, Satija R (2018). Integrating single-cell transcriptomic data across different conditions, technologies, and species. Nat. Biotechnol..

[9] Korsunsky I (2019). Fast, sensitive and accurate integration of single-cell data with Harmony. Nat. Methods.

[10] McInnes, L., Healy, J. & Melville, J. Umap: uniform manifold approximation and projection for dimension reduction. Preprint at https://arxiv.org/abs/1802.03426 (2018).

[11] Travaglini KJ (2020). A molecular cell atlas of the human lung from single-cell RNA sequencing. Nature.

[12] Aran D (2019). Reference-based analysis of lung single-cell sequencing reveals a transitional profibrotic macrophage. Nat. Immunol..

[13] Aibar S (2017). SCENIC: Single-cell regulatory network inference and clustering. Nat. Methods.

[14] Goldstraw P (2016). The IASLC lung cancer staging project: proposals for revision of the TNM stage groupings in the forthcoming (eighth) edition of the TNM classification for lung cancer. J. Thorac. Oncol..

[15] Patel AP (2014). Single-cell RNA-seq highlights intratumoral heterogeneity in primary glioblastoma. Science.

[16] Tirosh I (2016). Dissecting the multicellular ecosystem of metastatic melanoma by single-cell RNA-seq. Science.

[17] Tirosh I (2016). Single-cell RNA-seq supports a developmental hierarchy in human oligodendroglioma. Nature.

[18] Puram SV (2017). Single-cell transcriptomic analysis of primary and metastatic tumor ecosystems in head and neck cancer. Cell.

[19] Venteicher AS (2017). Decoupling genetics, lineages, and microenvironment in IDH-mutant gliomas by single-cell RNA-seq. Science.

[20] Cancer Genome Atlas Research N (2013). The cancer genome atlas pan-cancer analysis project. Nat. Genet..

[21] Paez JG (2004). EGFR: Mutations in lung cancer: correlation with clinical response to gefitinib therapy. Science.

[22] Kong LR (2020). A common MET polymorphism harnesses HER2 signaling to drive aggressive squamous cell carcinoma. Nat. Commun..

[23] Skinnider MA (2021). Cell type prioritization in single-cell data. Nat. Biotechnol..

[24] Efremova M, Vento-Tormo M, Teichmann, Vento-Tormo R (2019). CellPhoneDB v2.0: inferring cell-cell communication from combined expression of multi-subunit receptor-ligand complexes. Nat. Protoc..

[25] Myers KV, Amend SR, Pienta KJ (2019). Targeting Tyro3, Axl and MerTK (TAM receptors): implications for macrophages in the tumor microenvironment. Mol. Cancer.

[26] Chen HM (2018). Blocking immunoinhibitory receptor LILRB2 reprograms tumor-associated myeloid cells and promotes antitumor immunity. J. Clin. Investig..

[27] Yanai I (2005). Genome-wide midrange transcription profiles reveal expression level relationships in human tissue specification. Bioinformatics.

[28] Hwang DH (2016). KRAS and NKX2-1 mutations in invasive mucinous adenocarcinoma of the lung. J. Thorac. Oncol..

[29] Campbell JD (2016). Distinct patterns of somatic genome alterations in lung adenocarcinomas and squamous cell carcinomas. Nat. Genet..

[30] Pierce A (2008). Identification of a novel, functional role for S100A13 in invasive lung cancer cell lines. Eur. J. Cancer.

[31] Masso-Valles D, Beaulieu ME, Soucek L (2020). MYC, MYCL, and MYCN as therapeutic targets in lung cancer. Expert Opin. Ther. Targets.

[32] Farrell JA (2018). Single-cell reconstruction of developmental trajectories during zebrafish embryogenesis. Science.

[33] Shirasawa M (2004). Receptor for advanced glycation end-products is a marker of type I lung alveolar cells. Genes Cells.

[34] Newman GR, Campbell L, von Ruhland C, Jasani B, Gumbleton M (1999). Caveolin and its cellular and subcellular immunolocalisation in lung alveolar epithelium: implications for alveolar epithelial type I cell function. Cell Tissue Res..

[35] Christensen MV, Hogdall CK, Jochumsen KM, Hogdall EVS (2018). Annexin A2 and cancer: a systematic review. Int. J. Oncol..

[36] Jung HJ, Park JY, Jeon HS, Kwon TH (2011). Aquaporin-5: a marker protein for proliferation and migration of human breast cancer cells. PLoS ONE.

[37] Jamal-Hanjani M (2017). Tracking the evolution of non-small-cell lung cancer. N. Engl. J. Med..

[38] Parimon T, Yao C, Stripp BR, Noble PW, Chen P (2020). Alveolar epithelial type II cells as drivers of lung fibrosis in idiopathic pulmonary fibrosis. Int. J. Mol. Sci..

[39] Albertus DL (2008). AZGP1 autoantibody predicts survival and histone deacetylase inhibitors increase expression in lung adenocarcinoma. J. Thorac. Oncol..

[40] Parris TZ (2010). Clinical implications of gene dosage and gene expression patterns in diploid breast carcinoma. Clin. Cancer Res..

[41] Abbas W, Kumar A, Herbein G (2015). The eEF1A proteins: at the crossroads of oncogenesis, apoptosis, and viral infections. Front. Oncol..

[42] Liu S (2019). METTL13 methylation of eEF1A increases translational output to promote tumorigenesis. Cell.

[43] Rebecca VW (2017). A unified approach to targeting the lysosome’s degradative and growth signaling roles. Cancer Discov..

[44] Rebecca VW (2019). PPT1 promotes tumor growth and is the molecular target of chloroquine derivatives in cancer. Cancer Discov..

[45] Sharma G (2020). PPT1 inhibition enhances the antitumor activity of anti-PD-1 antibody in melanoma. JCI Insight.

[46] Christiansen A, Dyrskjot L (2013). The functional role of the novel biomarker karyopherin alpha 2 (KPNA2) in cancer. Cancer Lett..

[47] Huang JX, Wu YC, Cheng YY, Wang CL, Yu CJ (2019). IRF1 negatively regulates oncogenic KPNA2 expression under growth stimulation and hypoxia in lung cancer cells. Onco Targets Ther..

[48] Fu F (2020). Development and validation of a five-gene model to predict postoperative brain metastasis in operable lung adenocarcinoma. Int. J. Cancer.

[49] Valent P (2012). Cancer stem cell definitions and terminology: the devil is in the details. Nat. Rev. Cancer.

[50] Lathia JD, Liu H (2017). Overview of cancer stem cells and stemness for community oncologists. Target Oncol..

[51] Saygin C, Matei D, Majeti R, Reizes O, Lathia JD (2019). Targeting cancer stemness in the clinic: from hype to hope. Cell Stem Cell.

[52] Sloan AE (2014). Targeting glioma-initiating cells in GBM: ABTC-0904, a randomized phase 0/II study targeting the Sonic Hedgehog-signaling pathway. J. Clin. Oncol..

[53] Schott AF (2013). Preclinical and clinical studies of gamma secretase inhibitors with docetaxel on human breast tumors. Clin. Cancer Res..

[54] Vey N (2016). Phase I clinical study of RG7356, an anti-CD44 humanized antibody, in patients with acute myeloid leukemia. Oncotarget.

[55] Schepers K, Campbell TB, Passegue E (2015). Normal and leukemic stem cell niches: insights and therapeutic opportunities. Cell Stem Cell.

[56] Jones CL (2018). Inhibition of amino acid metabolism selectively targets human leukemia stem cells. Cancer Cell.

