# Supplementary material for: Integrated single-cell RNA sequencing analysis reveals distinct cellular and transcriptional modules associated with survival in lung cancer
Source: Signal Transduct Target Ther. 2022 Jan 14;7:9. doi: 10.1038/s41392-021-00824-9 (PMC8758688; doi:10.1038/s41392-021-00824-9)
Supplement: Supplementary file 1 — supplementary materials [file 41392_2021_824_MOESM1_ESM.docx]

Supplementary Materials for

Integrated Single-cell RNA Sequencing Analysis Reveals Distinct Cellular and Transcriptional Modules associated with survival in Lung Cancer

Li Zhang^1,5^, Yiming Zhang^2,5^, Chengdi Wang^1,5^, Ying Yang^1^, Yinyun Ni^1^, Zhoufeng Wang^1^, Tingting Song^1^, Menglin Yao^1^, Zhiqiang Liu^1^, Ningning Chao^1^, Yongfeng Yang^1^, Jun Shao^1^, Zhidan Li^2^, Ran Zhou^2^, Li Chen^2^, Dan Zhang^2^, Yuancun Zhao^2^, Wei Liu^2^, Yupeng Li^2^, Ping He^2^, Jing-wen Lin^2^, Yuan Wang^4^, Kang Zhang^3*^, Lu Chen^2*^, Weimin Li^1,6*^

Correspondence to: weimi003@scu.edu.cn (W. L.); luchen@scu.edu.cn (L. C.); kang.zhang@gmail.com (K. Z.)

**This PDF file includes:**

Materials and Methods

Supplementary Text

Figures. S1 to S7

Materials and Methods

**Sample Collection**

Fresh tumor tissues and adjacent normal tissues were collected from patients with primary lung cancer undergoing surgical resection without neoadjuvant therapy before surgery at West China Hospital (WCH). The disease stage was determined by the 8th edition of the American Joint Committee on Cancer (AJCC) TNM stage system ^14^. This study was approved by the Institutional Review Board of West China Hospital of Sichuan University (Chengdu China; Project identification code: 2017.114 and 2018.270) and all patients signed informed written consent. Clinical characteristics including age, sex, smoking status, pathological subtype and stage were recorded at recruitment listed in Supplementary Table S1.

**Preparation of single-cell suspensions**

Freshly obtained resected tissues were rinsed with HBSS after the operation, subsequently shredded on ice to smaller pieces with collagenase I/IV in HBSS, and incubated for 30min at 37 ℃ with manual shaking every 5 minutes. The digested tissues were then passed through a 40-μm nylon mesh filter, and suspended cells were centrifuged at 500 g for 5 minutes. After removing the supernatant, the pelted cells were suspended in red blood lysis buffer, and next resuspended in sorting buffer (0.04% BSA + PBS) after washing with HBSS. Cell suspensions, after depleting dead cells through flow cytometry, were directly processed for single-cell RNA-seq, following the manufacturer’s instructions. Alternatively, cell suspensions were frozen in 20% Dimethyl Sulfoxide (DMSO) and Fetal Bovine Serum (FBS).

**Hematoxylin and eosin (H&E) staining**

Surgically resected tissues were fixed in 10% neutral formalin solution for 48-72 hours. All tissues were graded dehydrated and immersed in wax for 15 hours. The 3 to 5μm sections were baked at 67 °C for 4 h after the paraffin embedding and deparaffinized in xylene and graded ethanol in distilled water. Cell nuclei were counterstained with hematoxylin for 3 min and washed with deionized distilled water. All samples were stained with eosin for 30 s-1 min, followed by gradient alcohol dehydration, xylene transparent, and final neutral gum sealing. The images were collected by an Olympus IX83 microscope.

**Immunofluorescence (IF) microscopy**

Surgically resected tissues were immediately fixed in 10% neutral formalin solution for 48-72 hours. All tissues were graded dehydrated and immersed in wax as the following routine methods ^57^. The 3 to 5 μm sections were baked at 67 °C for 4 h after the paraffin embedding, then sections were deparaffinized in xylene and graded ethanol in distilled water and were deparaffinized in xylene and graded ethanol in distilled water. Antigen retrieval was performed with microwave in a water bath by Tris-EDTA solution for 3-8 min. After blocking endogenous peroxides for 15 minutes at room temperature by 3% H_2_O_2_, all sections were then blocked with goat serum for 1 h at RT and incubated with primary antibodies overnight at 4 °C. Primary antibodies were visualized by species-specific goat secondary antibodies conjugated to Alexa Fluor dyes (Alexa 488/555, 1:400, Jackson) for 1 h at RT. Sections were then stained with DAPI (1μg/ml) for 5 min. Slides were cover-slipped and imaged under an Olympus IX83 fluorescent microscope. The antibodies used in the experiments were：AQP5 (Aquaporin 5, abcam, ab92320), S100A13 (abcam, ab109252), AZGP1 (proteintech,13399-1-AP), ANXA1 (proteintech, 21990-1-AP), ATF3 (abcam, ab254268), EEF1A2 (proteintech, 16091-1-AP), CHI3L1 (abcam, ab255297), KPNA2 (proteintech, 10819-1-AP), PPT1 (GeneTex, GTX110677), SPB (Santa, sc-133143), AGER (R&D, AF1145), KRT5 (ZEN BIO, 252857), CK5/6 (Millpore, MAB1620).

***In vitro* overexpression experiments**

Human lung cancer cell line H1299 was obtained from the American Type Culture Collection and was genotyped and authenticated before experiments. H1299 were cultured in RPMI-1640 medium (HyClone) supplemented with 10% fetal bovine serum (ZETA) at 37 °C in a humidified incubator with 5% CO2. Overexpression and control plasmids were synthesized by GeneCopoeia. Plasmids were transfected with lipofectamine 3000 (Invitrogen) reagent at a concentration of 1 μg/ml.

**Assay of cell viability, migration and invasion**

Overexpression and control cells were plated into 96-well plates (1×10^4^ cells/well) 24-hour post-transfection. CCK-8 reagent (ZETA) was added after 48 h cultivation and absorbance was detected at 450 nm with a microplate reader (BioTek, Winooski, VT). Detections of migration and invasion were performed by 24-well transwell inserts (8 μm pore size, Millipore, USA). For migration assays, a medium containing 10% FBS was added to the lower chamber, and 2×10^4^ transfected cells in serum-free medium were cultured in the upper chamber. For invasion experiments, Matrigel mix (BD Biosciences, CA, USA) was pre-coated the top chamber of transwell inserts and 4×10^4^ transfected cells were seeded in the top chamber. After 48 h, cells invaded to the underside of the membrane were fixed with methanol and stained with 0.1% crystal violet for 30 min at RT, imaged and counted with a microscope (Leica, Wetzlar, Germany) and Olympus cellSens standard software (v1.5).

## Quantification And Statistical Analysis

**Single-cell RNA sequencing and analysis**

*Library preparation and sequencing.* Single cells were prepared in the Chromium Single Cell Gene Expression Solution using the Chromium Single Cell 3′ Gel Bead, Chip and Library Kits v2 (10X Genomics) as per the manufacturer’s protocol. 8000-10000 total cells were added to each channel. The cells were then partitioned into Gel Beads in Emulsion in the Chromium instrument, where cell lysis and barcoded reverse transcription of RNA occurred, followed by amplification, shearing and 5′ adaptor and sample index attachment. Libraries were sequenced on the Illumina NovaSeq 6000 platform in West China Hospital, Chengdu.

*Alignment and quantification.* We generated 52 samples from 32 individuals and downloaded raw fastq files from a previous publication with 3 LUAD and 3 LUSC samples ^5^. Gene expression matrices were generated per sample using CellRanger (v3.0.1), and converted to a Seurat object using the Seurat R package (v2.3.4) ^8^. Samples with less than 500 cells were removed. Cells were required to have more than 1000 UMIs and only genes with more than 1000 UMIs across all cells were kept for further analyses.

*Clustering and annotation.* For the remaining 293,432 cells after quality control, gene expression matrices were normalized by library size, and scaled after correcting technical covariates (total cellular read count and mitochondrial read count)*.* To reduce the dimensionality of this dataset, the resulting 1,981 variably expressed genes were summarized for principal component (PC) analysis. The statistically significant PCs were used for Harmony (v0.99.9) ^9^ to remove the batch effect, and the two-dimension UMAP was calculated among the first 18 Harmony matrix by RunUMAP with default parameters. Then the original Louvain algorithm (FindClusters) with clustering resolution 0.8 was performed to cluster the cells. Cell clusters in the resulting two-dimensional representation were annotated to known biological cell types using canonical marker genes.

*Cell type classification and cell composition module construction.* AUC scores were calculated by AUCell (v1.6.1) ^13^ based on cell markers collected from previous studies ^5^. And we manually curated the threshold of the AUC score to separate different clusters into specific cell types. To refine the classification of immune cell types, we further compared our cell-type annotation to Blueprint reference (**Figure S1**E and F) using SingleR (v1.0.1) ^12^.

Cell composition was calculated using the number of cells of each type divided by the total number of cells in this sample. Pearson and Spearman correlation was then used to assess the distance and cluster all the samples. To assess whether the cell composition can distinguish different tissue types among normal, LUAD and LUSC, we compared various machine learning methods in which the AUC values were calculated using SVM from scikit-learn (v0.22.2) ^58^ achieved the highest accuracy. To prioritize cell types, we used ‘calculate_auc’ from Augur (v1.0.2) ^23^ comparing LUAD, LUSC, adjacent samples of LUAD and adjacent samples of LUSC with each other. Survival analysis was performed to compare the samples with different cell compositions using TCGABiolinks ^59,60^. For survival analyses, the Log-rank test was used to determine the differences between Kaplan-Meier survival curves.

*Copy Number Alteration (CNA).* CNAs of each cell type were estimated by sorting the analyzed genes on chromosomal location and applying a moving average to the relative expression values, with a sliding window of 100 genes within each chromosome by inferCNV (v1.0.4) ^15-19^. Then we combined the K-means clustering algorithm and UMAP reduction to separate all cells into multiple subclones, and the non-malignant cells were defined as the cells with the least CNVs.

*Inferring cell-cell communication networks.* We performed CellPhoneDB (v2.0.0) ^24^, a manually curated repository of ligands, receptors and their interactions, to predict cell-cell communication networks from single-cell transcriptome data. Only cell type-specific receptors and ligands expressed in more than 30% of the cells in the specific cluster were enrolled in the analysis.

*Identification of marker genes and stage module construction*. To identify cell-type marker genes, we used the Seurat FindMarkers function. Only differentially expressed (log2 fold-change > 0.25, adjust p-value < 0.05) and highly expressed in 50% of cells from the cluster were used. To identify the stage-specific genes, we used FindMarkers to compare cells from different stages in LUAD and LUSC. Marker genes were required to have at least 0.5-fold higher than the average expression in other stages. Furthermore, the Mfuzz (v2.44.0) ^61^ was used to filter and classify genes to each stage.

*Transcription factor-target gene network analysis*. Core regulatory transcription factors were predicted based on the scRNA-seq data. We constructed pseudo-bulk samples by adding up all the single cells from the same cell type and then calculated the mean expression level of each gene. The TF regulatory network was then computed by LeMoNe (v2.5) ^62^ based on these gene expressions.

*Subclustering of the major cell types.* We applied dimensionality reduction using harmony analysis in each cell type on variably expressed genes as described above. To identify which principal components were informative, we applied the kneedle algorithm ^63^ as implemented in the Python module kneed (v0.5.0) to the standard deviation of different components. Using the graph-based clustering approach implemented in the FindClusters function of the Seurat package, with a conservative resolution of 0.1 and otherwise default parameters. For visualization purposes, these informative principle components were converted into UMAP plots. To further explore the characteristics of different clusters, the cluster marker genes were identified using FindMarkers (log fold change of average expression > 0.25 and expressed in > 10% of all cells from that cluster).

*Trajectory analysis.* We first detected highly variable genes for each cluster and calculated the diffusion map with the default parameters, then inferred the pseudo-time using stage I as the root cells. Differential expression tests along the pseudo-time were performed by URD (v1.1.0) ^32^.

*Cell cycling analysis*. The cell cycle phase scores were calculated based on canonical markers using Seurat (v3.1.0) ^64^. The G1 phase was defined as Non-Cycling, and G2M and S were defined as a cycling state. The cluster score of each cell was calculated using the mean level expression of the top 50 cluster markers.

**Integrations of published datasets**

*TCGA bulk RNA-seq and copy number variation Dataset*. We analyzed TCGA data ^20^ retrieved using TCGABiolinks (v2.12.6) ^59,60^. Specifically, we downloaded gene expression data (raw counts) as well as clinical data for primary solid tumors and normal solid tissue, for LUAD (TCGA-LUAD) and LUSC (TCGA-LUSC). We then used GAIA (v2.28.0) ^65^ to infer the recurrent CNVs. Next, the aberrant recurrent genomic regions were annotated using findOverlaps from GenomicRanges (v1.36.1) ^66^. The aberrant genomic regions were visualized by circlize (v0.4.8) ^67^.

*East Asians, GTEx and CHOICE bulk RNA-seq Dataset.* The raw counts of East Asians were downloaded from OncoSG (<https://src.gisapps.org/OncoSG_public/datasets>) under dataset “Lung Adenocarcinoma (GIS, 2019)”^6^. The raw counts of normal lung tissue (release v8) were downloaded from the GTEx portal (<https://gtexportal.org/home/>) ^68^. The RNA-seq FPKM data from CHOICE were retrieved from figureshare (<https://doi.org/10.6084/m9.figshare.7306364.v1>) ^69^.

*West-china Hospital WES, Bulk RNA-seq and ATAC-seq Dataset.* We analyzed our previously published 45 RNA-seq expression proﬁles of LUAD, LUSC and BSPN (<https://pms.cd120.com>) ^7^. We removed RNA-seq sample LUAD50 and ATAC-seq LUAD10 from downstream analyses due to low quality. These bulk RNA-seq were aligned to human genome hg38 (Ensembl 93) by STAR (v2.6.1a) ^70^. Gene expression was quantified using RSEM (v1.3.1) ^71^ with default parameters. Then the differential expressed genes were calculated by DESeq2 (v1.24.0) ^72^. The regions of somatic CNV were leftovers to hg38 genome using LiftOver (http://hgdownload.cse.ucsc.edu/admin/exe/) and annotated with gene using findOverlaps from GenomicRanges (v1.36.1) ^66^. The aberrant genomic regions were visualized using circlize (v0.4.8) ^67^. Then the hg19 version of ATAC-seq narrows peaks, bam files and promoter regions annotated by TxDb. Hsapiens.UCSC.hg19.knownGene (v3.2.2) ^73^ was taken as the input of DiffBind (v2.12.0) ^74^ for downstream ATAC-seq differential expression analysis.

*Deconvolution*. The raw counts of scRNA-seq with log2 transformed were used to calculate the specificity index (tau value). Cell type-specificity index tau was defined previously ^27^ as:

$$\tau= \frac{\sum_{i=1}^{N} (1- x_{i})}{N-1}$$

Where N is the number of tissues or cell types, and x_i_ is the expression profile component normalized by the maximal component value.

Then the high cell type-specific genes (tau > 0.9) and cell markers were used as a reference, and the deconvolution algorithm MuSiC (v0.1.1) ^75^ and dtangle (v0.3.1) ^76^ were used to evaluate the proportion of different cell types in each sample.

*GO and DOSE analysis.* GO biological process and pathway enrichment analysis was performed using enrichGO and compareCluster from clusterProfiler ^77^. The disease ontology enrichment analyses were performed using enrichDO from clusterProfiler with default parameters. Results were visualized with the ggplot2 ^78^.

**Statistical analyses**

All statistical analyses were performed using R (v3.6.0) and GraphPad Prism (v8.3.1). GraphPad Prism software was used to conduct the Student’s t-tests for cell viability, migration and invasion.

Supplementary Text

**Data and code availability**

All the raw data of scRNA-seq have been deposited in the <https://pms.cd120.com/wcd01/index.html>. All raw counts, TPM matrix, the Seurat object including expression matrix and cell annotation information of single-cell RNA-seq are available in <http://lungcancer.chenlulab.com> (**Figure S7**e). The code is available at <https://github.com/LuChenLab/inferCC>.

**Acknowledgements**

This study was funded by the National Natural Science Foundation of China (grants 81974363, 81772478, 81871890, and 91859203), the National Key Research and Development Program of China, Stem Cell and Translational Research (2017YFA0106800 and 2017YFA0106500) and the National Science Fund for Excellent Young Scholars (81722004) to L.C.

**Author contributions**

W.L., L.C., and K.Z. conceived the project and designed the experiments. L.Z., Y.Z., and C.W., carried out experiments and performed bioinformatic analysis. Y.Yang., Y.N., Z.W., T.S., M.Y., Z.L., N.C., Y.Y., Z.L., R.Z., L.C., D.Z., Y.Z., W.Liu., Y.L., and Q.X., contributed to the experiments and analyzed the data. All authors discussed the results and reviewed the manuscript.

**Conflicts of interests**

The authors declare no competing interests.


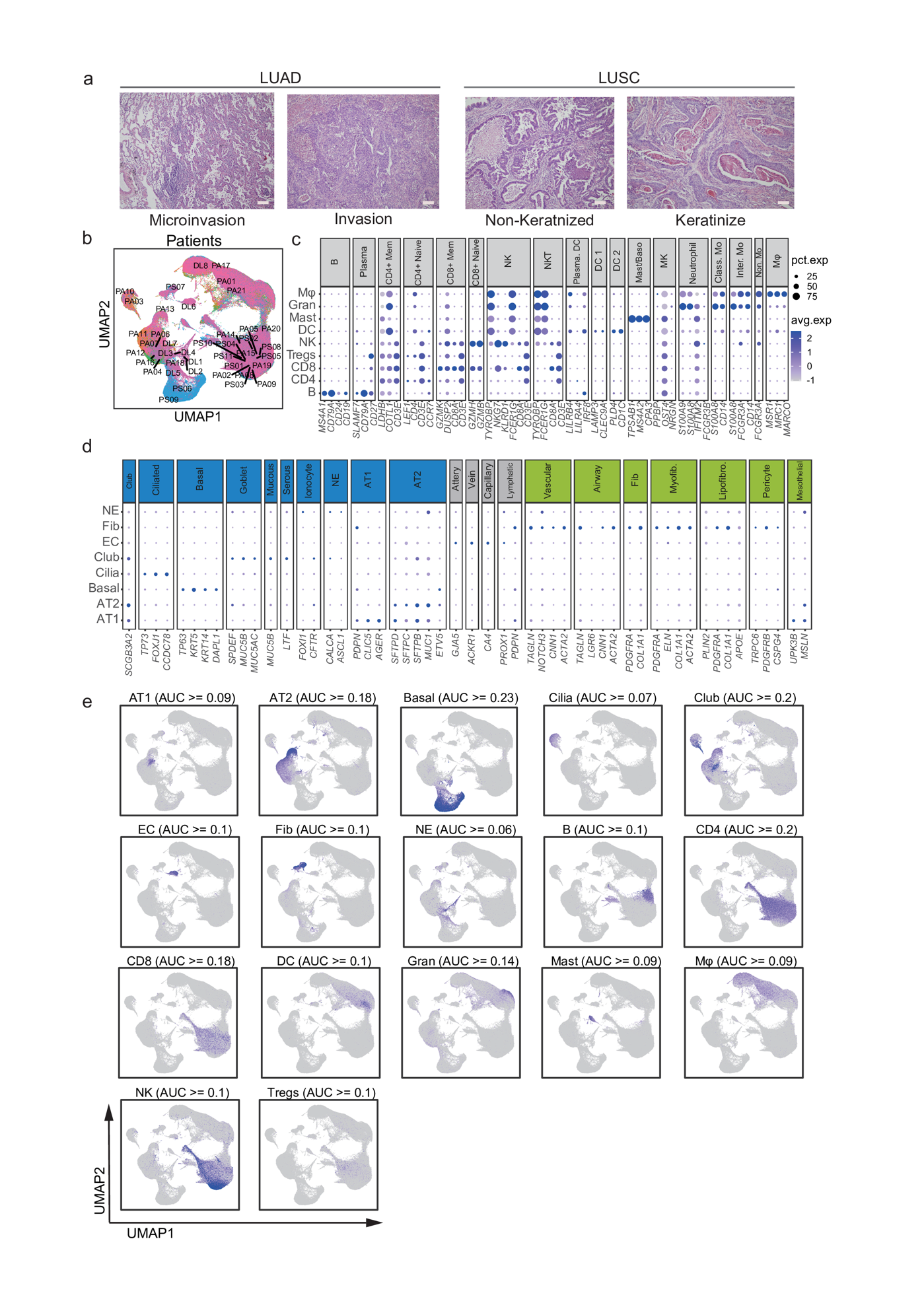


Figure. S1. related to Figure 1.

(**a**) Hematoxylin and eosin (HE) stain of LUAD and LUSC, scale bar, 100μm.

(**b**) UMAP of the 293,432 cells color by the corresponding patient.

(**c-d**) Dot plot showed the expression level of cell type-specific marker genes for immune (c) and non-immune (d) cells. The epithelial cells (green), the endothelial cells (grey), and stromal cells (blue) were color-coded. The dot size represents the fraction of cells expressed corresponding genes, and the intensity of dot color represented the average expression value. (**e**) UMAP plots of AUC score for cell type-specific gene sets that calculated using expression data for 17 cell types using AUCell.


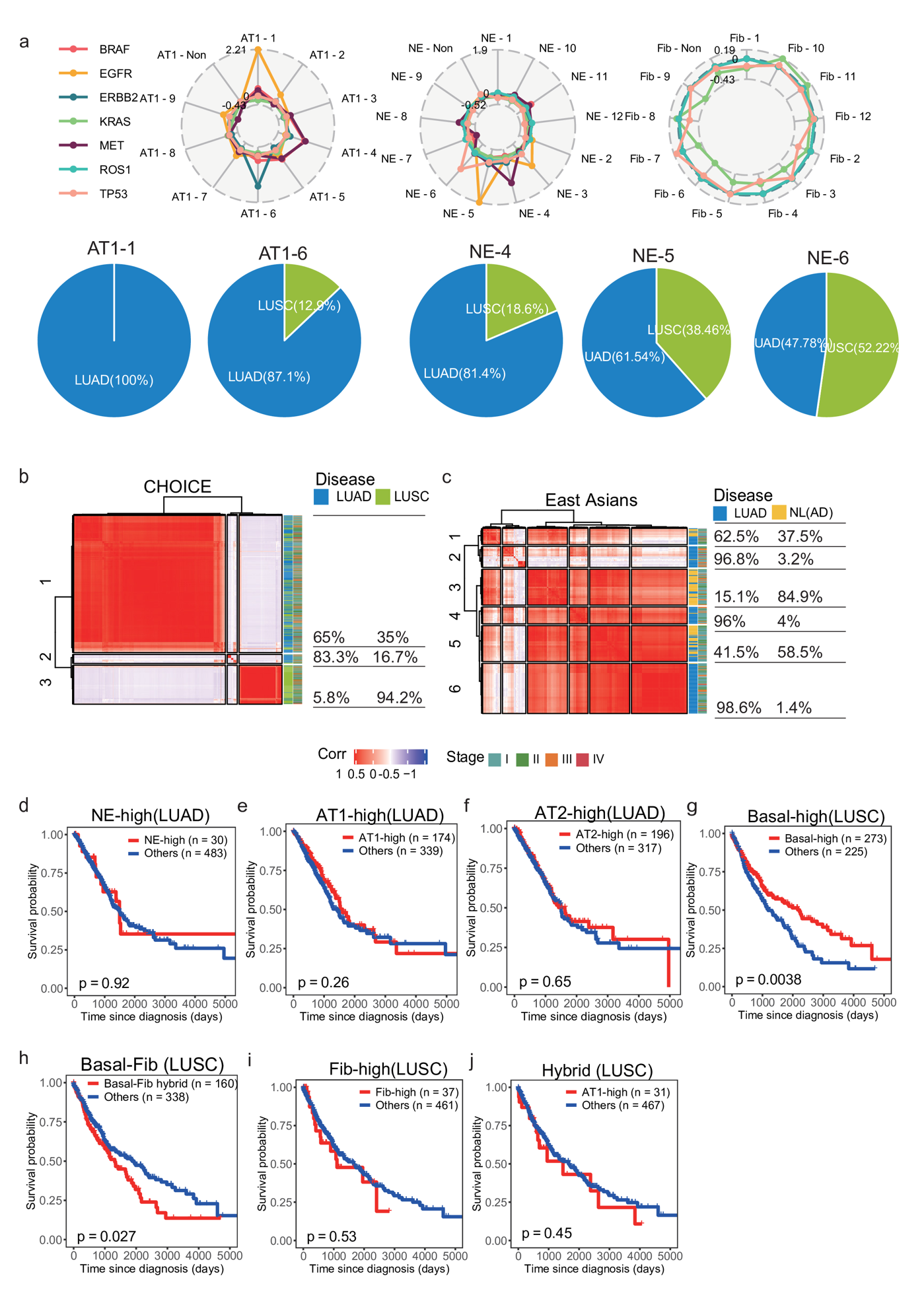


Figure. S2. related to Figures 2 and 3.

(**a**) Radar plots illustrate the average inferred CNV score of the driver-genes in subtypes of AT1, NE, fibroblast and macrophage (left to right). Pie charts presented the percentage of cells from LUSC or LUAD.

(**b-c**) The Pearson correlation between cell weights of samples from CHOICE (b) and East Asians (c).

(**d-f**) Kaplan-Meier survival curves for patients in LUAD. Log-rank test p values were shown after correcting for age, gender, study and stage in NE-high (d), AT1-high (e) and AT2-high (f). P-value was calculated using the log-rank test.

(**g-j**) Kaplan-Meier survival curves for patients in LUSC. Log-rank test p values were shown after correcting for age, gender, study and stage in basal-high (g), basal-fib hybrid (h), fib-high (i), and hybrid (j). P-value was calculated using the log-rank test.


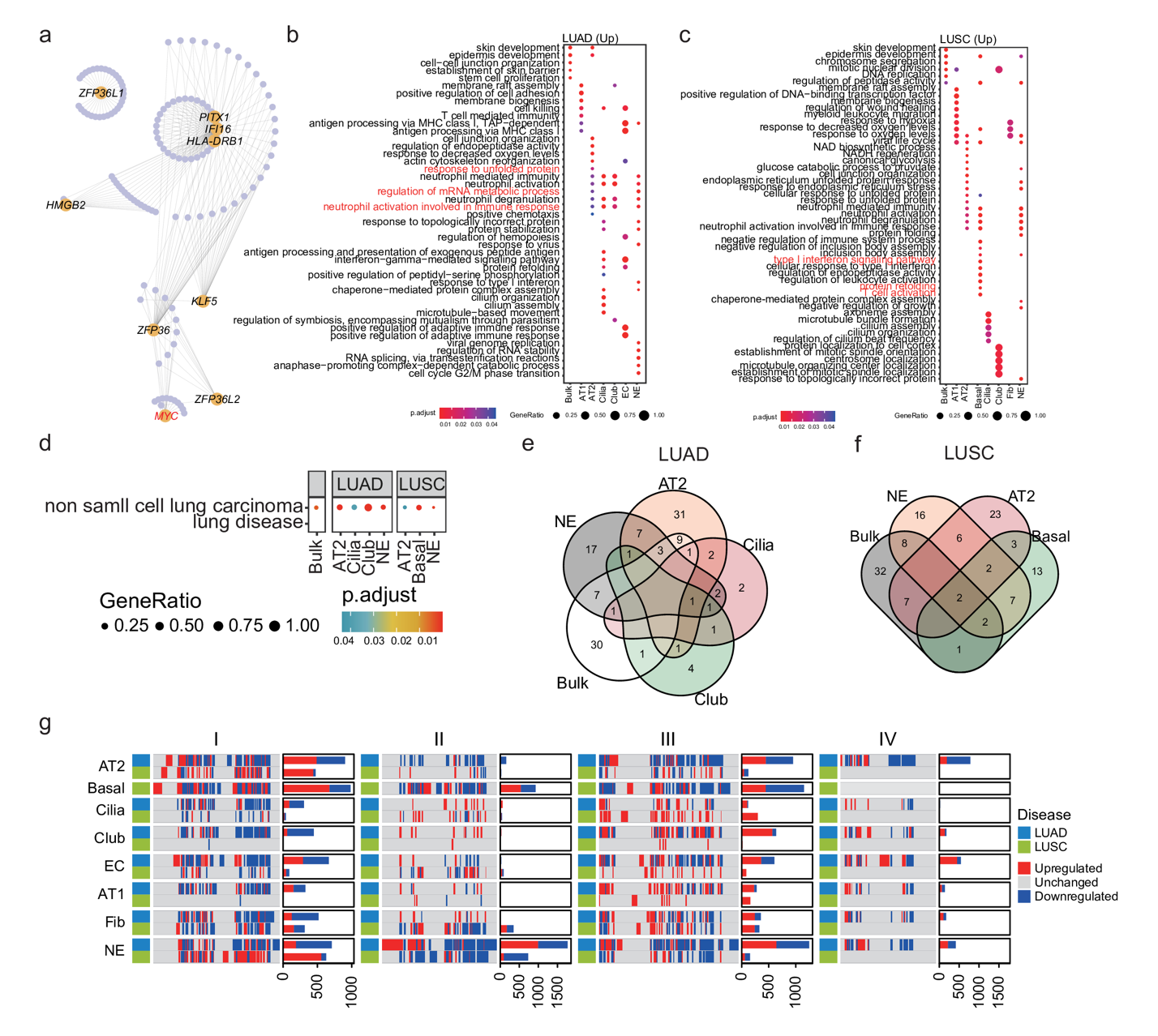


Figure. S3. related to Figure 5.

(**a**) The regulatory networks of upregulated DEGs of basal from LUSC. Only the top regulators identified by LeMoNe were drawn.

(**b-c**) GO enrichment analysis of the up-regulated genes in bulk RNA-seq and 7 cell types of LUAD (**b**) and LUSC (**c**).

(**d**) Representative Disease Ontology (DO) terms and pathways enriched in upregulated DEGs based on functional enrichment analysis (p < 0.05).

(**e-f**) Venn diagrams showed the numbers of genes enriched in non-small cell lung carcinoma pathway in each cell type and bulk sample from LUAD (e) and LUSC (f).

(**g**) Distribution of stage-specific genes in 7 cell types and bulk RNA-seq. Each row represented one cell type in a specific lung cancer subtype, and each column represented one gene. Red, upregulated (average logFC > 0.25 for scRNA-seq and logFC > 1 for bulk RNA-seq, adjusted p-value < 0.05); blue, downregulated (average logFC < -0.25 for scRNA-seq and logFC < -1 for bulk RNA-seq, adjusted p-value < 0.05); gray, unchanged (|average logFC| < 0.25 for scRNA-seq and |logFC| < 1 for bulk RNA-seq).


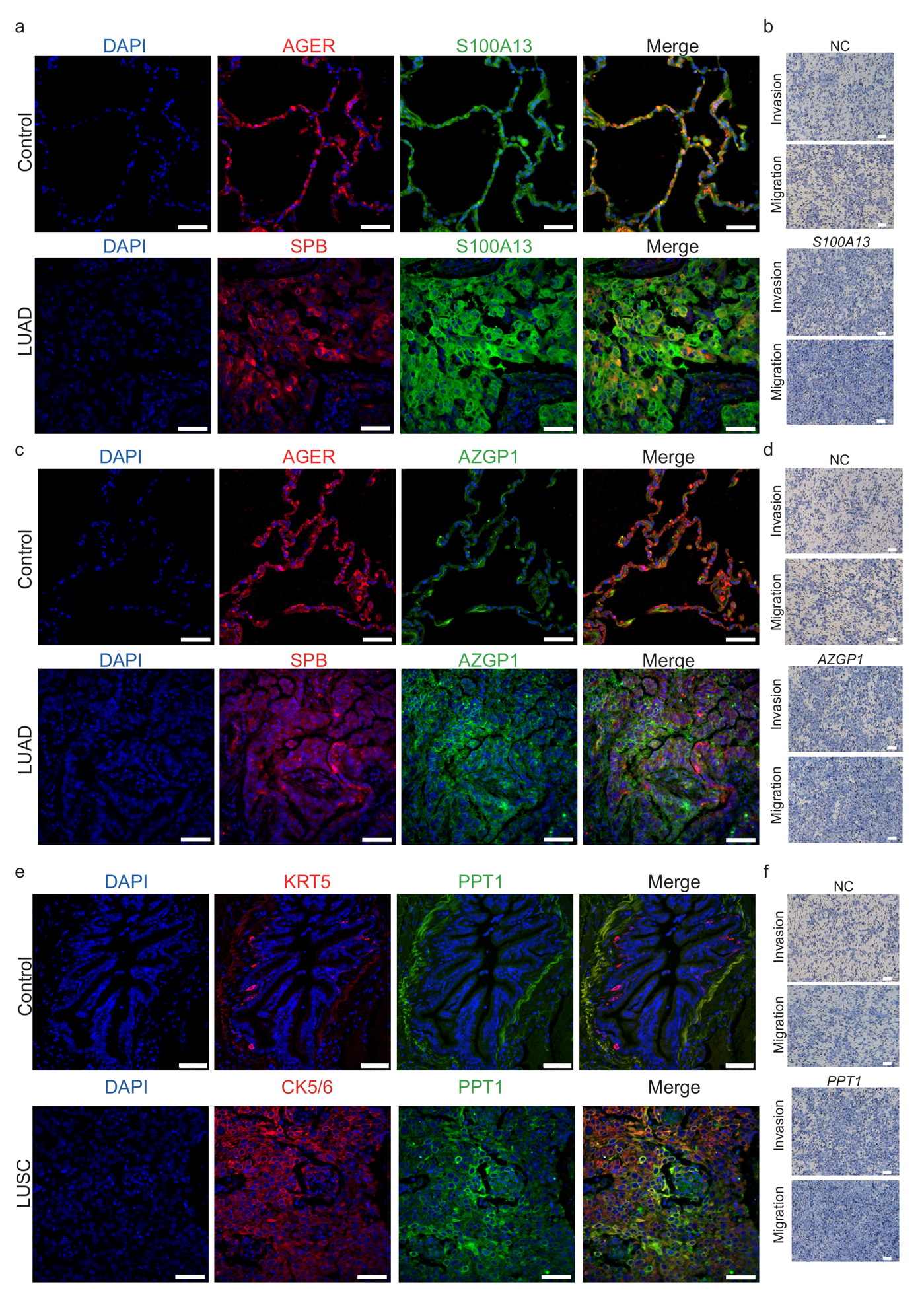


**Figure. S4. related to Figure 5.**

**(a)** The expression of *S100A13* in LUAD. Immunofluorescence staining indicated the location of *S100A13* level in lung cancer cells (green), SPB was the marker of lung adenocarcinoma cells (red); AGER was the marker of AT1 cells in normal lung. The cell nucleus was co-stained with DAPI (blue); Scale bar, 50μm.

**(b)** The invasion and migration of negative control (NC）and *S100A13* overexpression H1299 cells. Transwell assays were conducted for cell migration (without matrigel) and invasion abilities (with matrigel). Scale bar, 100μm.

**(c)** The expression of *AZGP1* in LUAD. Immunofluorescence staining indicated the location of *AZGP1* level in lung cancer cells (green), SPB was the marker of lung adenocarcinoma cells (red); AGER was the marker of AT1 cells in normal lung. The cell nucleus was co-stained with DAPI (blue); Scale bar, 50μm.

**(d)** The invasion and migration cells of negative control (NC）and *AZGP1* overexpression H1299 cells. Transwell assays were conducted for cell migration (without matrigel) and invasion abilities (with matrigel). Scale bar, 100μm.

**(e)** The expression of *PPT1* in LUSC. Immunofluorescence staining indicated the location of *PPT1* level in lung cancer cells (green), CK5/6 was the marker of lung squamous carcinoma cells (red), KRT5 was the marker of basal cells in normal lung. The cell nucleus was co-stained with DAPI (blue); Scale bar, 50μm.

**(f)** The invasion and migration cells of negative control (NC）and *PPT1* overexpression H1299 cells. Transwell assays were conducted for cell migration (without matrigel) and invasion abilities (with matrigel). Scale bar, 100μm.


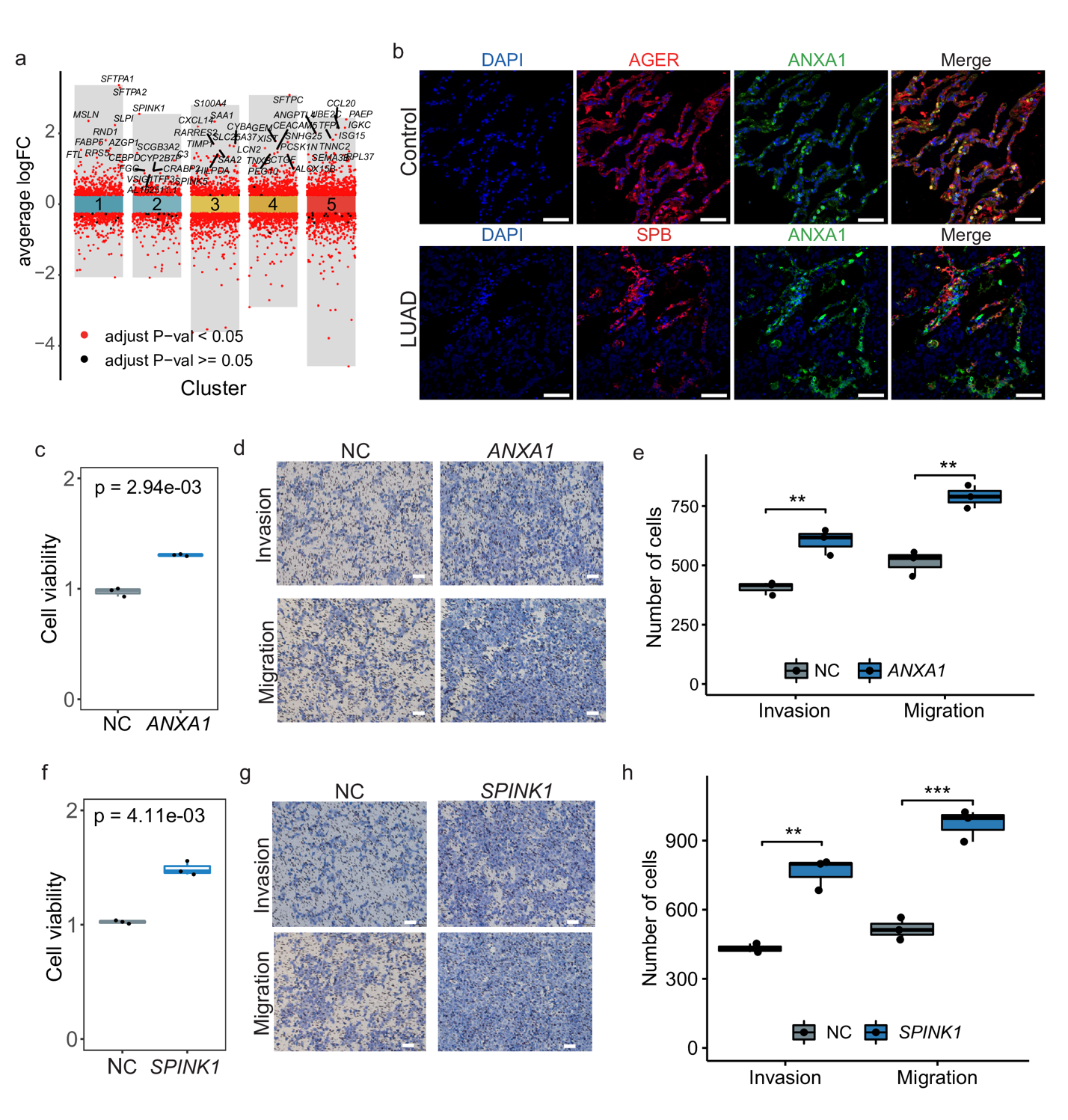


**Figure S5. related to Figure 6.**

**(a)** The scatter plot showed the cluster-specific genes of AT2 from LUAD. The top 10 specific genes were labeled.

(**b**) The expression of *ANXA1* in LUAD. Immunofluorescence staining indicated the location of *ANXA1* level in lung cancer cells (green), SPB was the marker of lung adenocarcinoma cells (red); AGER was the marker of AT1 cells in normal lung. The cell nucleus was co-stained with DAPI (blue); Scale bar, 50μm.

(**c**) The cell viability of *ANXA1* overexpression H1299 cells. Cell viability detection was completed by CCK8 detection. The p-value was calculated using t test.

(**d**) The invasion and migration of *ANXA1* overexpression H1299 cells. Transwell assays were conducted for cell migration (without matrigel) and invasion abilities (with matrigel). Scale bar, 100μm.

(**e**) The boxplot showed the number of invasion and migration cells of *ANXA1* overexpression H1299 cells. The p value was calculated by the Student’s t-test.

(**f**) The cell viability of *SPINK1* overexpression H1299 cells. Cell viability detection was completed by CCK8 detection. The p-value was calculated using t test.

(**g**) The invasion and migration of *SPINK1* overexpression H1299 cells. Transwell assays were conducted for cell migration (without matrigel) and invasion abilities (with matrigel). Scale bar, 100μm.

(**h**) The boxplot showed the number of invasion and migration cells of *SPINK1* overexpression H1299 cells. The p value was calculated by the Student’s t-test.

(**i**) The expression of *CHI3L1* in LUAD. Immunofluorescence staining indicated the location of *CHI3L1* level in lung cancer cells (green), SPB was the marker of lung adenocarcinoma cells (red); AGER was the marker of AT1 cells in normal lung. The cell nucleus was co-stained with DAPI (blue); Scale bar, 50μm.

(**j**) The cell viability of *CHI3L1* overexpression H1299 cells. Cell viability detection was completed by CCK8 detection. The p-value was calculated using t test.

(**k**) The invasion and migration of *CHI3L1* overexpression H1299 cells. Transwell assays were conducted for cell migration (without matrigel) and invasion abilities (with matrigel). Scale bar, 100μm.

(**l**) The boxplot showed the number of invasion and migration cells of *CHI3L1* overexpression H1299 cells. The p value was calculated by the Student’s t-test.


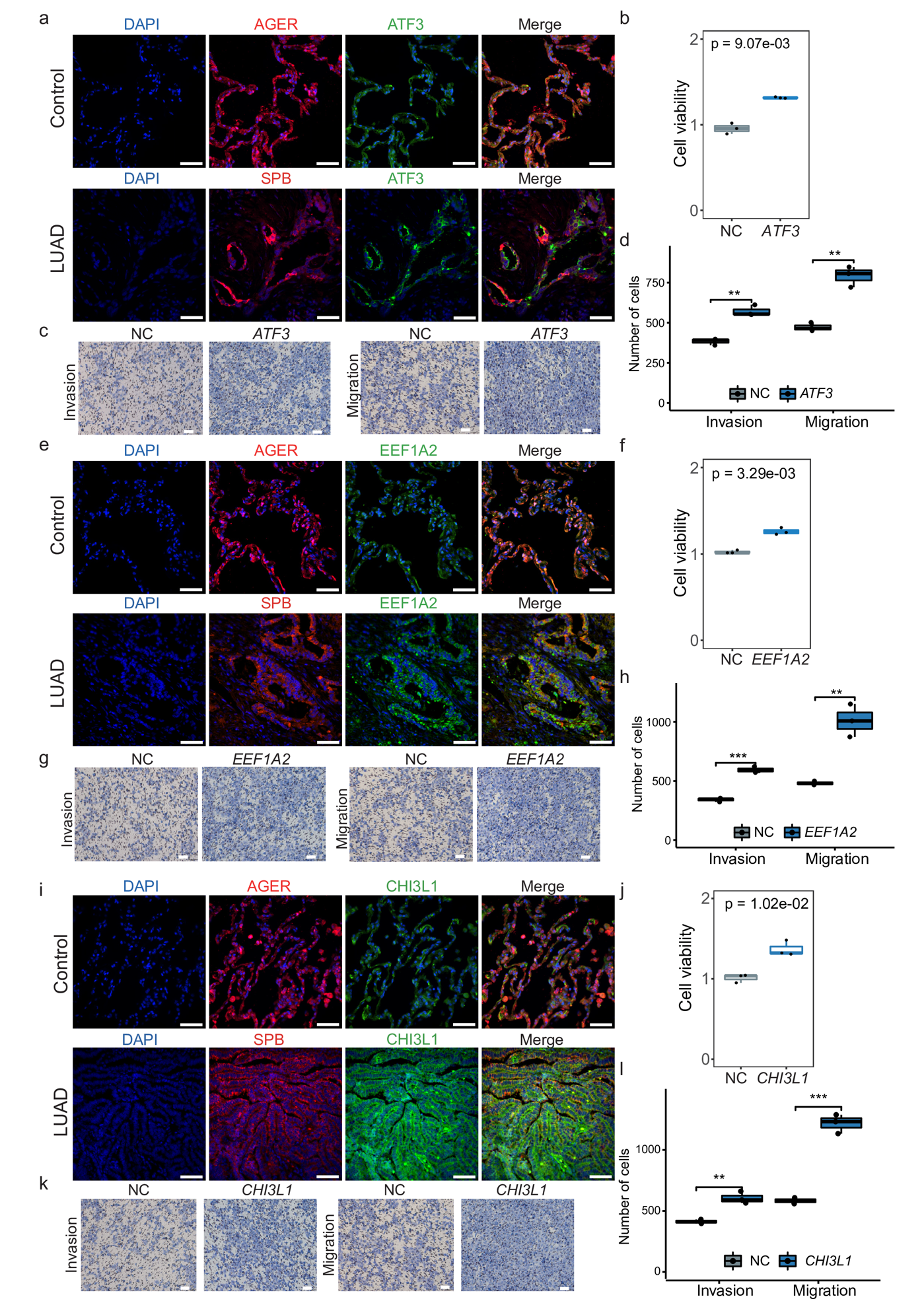


**Figure. S6. related to Figure 6.**

(a) The expression of *ATF3* in LUAD. Immunofluorescence staining indicated the location of *ATF3* level in lung cancer cells (green), SPB was the marker of lung adenocarcinoma cells (red); AGER was the marker of AT1 cells in normal lung. The cell nucleus was co-stained with DAPI (blue); Scale bar, 50μm.

(**b**) The cell viability of *ATF3* overexpression H1299 cells. Cell viability detection was completed by CCK8 detection. The p-value was calculated using t test.

(**c**) The invasion and migration of *ATF3* overexpression H1299 cells. Transwell assays were conducted for cell migration (without matrigel) and invasion abilities (with matrigel). Scale bar, 100μm.

(**d**) The boxplot showed the number of invasion and migration cells of *ATF3* overexpression H1299 cells. The p value was calculated by the Student’s t-test.

(**e**) The expression of *EEF1A2* in LUAD. Immunofluorescence staining indicated the location of *EEF1A2* level in lung cancer cells (green), SPB was the marker of lung adenocarcinoma cells (red); AGER was the marker of AT1 cells in normal lung. The cell nucleus was co-stained with DAPI (blue); Scale bar, 50μm.

(**f**) The cell viability of *EEF1A2* overexpression H1299 cells. Cell viability detection was completed by CCK8 detection. The p-value was calculated using t test.

(**g**) The invasion and migration of *EEF1A2* overexpression H1299 cells. Transwell assays were conducted for cell migration (without matrigel) and invasion abilities (with matrigel). Scale bar, 100μm.

(**h**) The boxplot showed the number of invasion and migration cells of *EEF1A2* overexpression H1299 cells. The p value was calculated by the Student’s t-test.


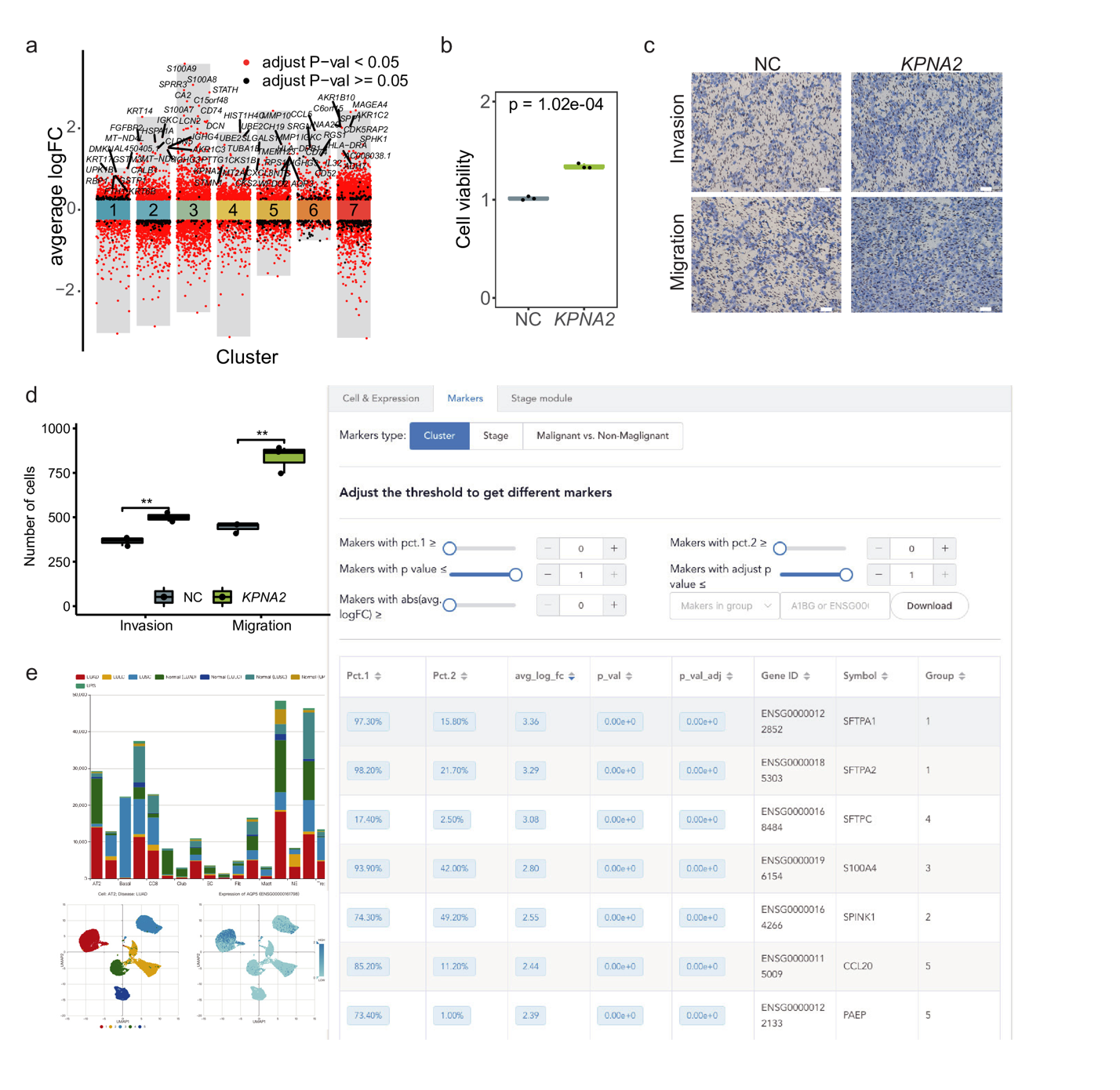


**Figure. S7. related to Figure 7.**

**(a)** The scatter plot showed the cluster-specific genes of basal from LUSC. The top 10 specific genes were labeled.

**(b)** The cell viability of *KPNA2* overexpression H1299 cells. Cell viability detection was completed by CCK8 detection. The p-value was calculated using t test.

**(c)** The invasion and migration of *KPNA2* overexpression H1299 cells. Transwell assays were conducted for cell migration (without matrigel) and invasion abilities (with matrigel). Scale bar, 100μm.

**(d)** The boxplot showed the number of invasion and migration cells of *KPNA2* overexpression H1299 cells. The p value was calculated by the Student’s t-test.

(**e**) The main components of our website, including a bar plot showed the number of cells by sample origin, patients. An interactive scatter plot showed the UMAP distribution of malignant cells by cell type. An interactive scatter plot showed the expression levels of chosen genes. Table with multiple control elements for users to explore and download related information.
